# Engineering de novo formatotrophy in the non-model yeast *Y. lipolytica*

**DOI:** 10.1038/s44160-026-01076-7

**Published:** 2026-06-12

**Authors:** William Newell, Piotr Hapeta, David Bell, Rodrigo Ledesma-Amaro

**Affiliations:** 1 Department of Bioengineering and Imperial College Centre for Engineering Biology, Imperial College London, London, UK; 2 Bezos Centre for Sustainable Protein, Imperial College London, London, UK; 3 UKRI Engineering Biology Mission Hub on Microbial Food, Imperial College London, London, UK; 4 London Biofoundry, Imperial College London, London, UK

**Keywords:** Industrial microbiology, Synthetic biology

## Abstract

Formate is an exciting potential microbial feedstock as it can be derived from CO_2_ and electricity. Despite this, limited progress has been made in engineering formatotrophy in yeasts, and no yeasts grow using formate naturally. Here we use metabolic modelling to find two potential formatotrophy pathways in *Yarrowia lipolytica*. We then use C13 tracer analysis and computationally guided growth experiments to show that wild-type *Y. lipolytica* possesses strong formate dissimilation and a cyclical C1 pathway with similar architecture to the synthetic serine–threonine cycle, which it uses to co-assimilate formate and glycerol. Messenger RNA sequencing shows that formate exposure results in increased oxidative stress and changes in the tricarboxylic acid cycle, redox and C1 metabolism. Following this, we use model-guided adaptive laboratory evolution to produce a formatotrophic strain of *Y. lipolytica* using the eukaryotic serine–threonine cycle. We then use further messenger RNA sequencing to show that formatotrophy is supported by changes in adenosine triphosphate and reactive oxygen species metabolism. Subsequently, we engineer nicotinamide adenine dinucleotide phosphate (NADPH) and reactive oxygen species metabolism to create a strain with substantially improved growth. This strain reaches about 10% of the theoretical maximum biomass yield, highlighting its potential for additional engineering approaches. Finally, we show that beta-carotene production from formate is possible in our engineered strain, opening the door to formatotrophic eukaryote bioprocesses.

**Figure:**
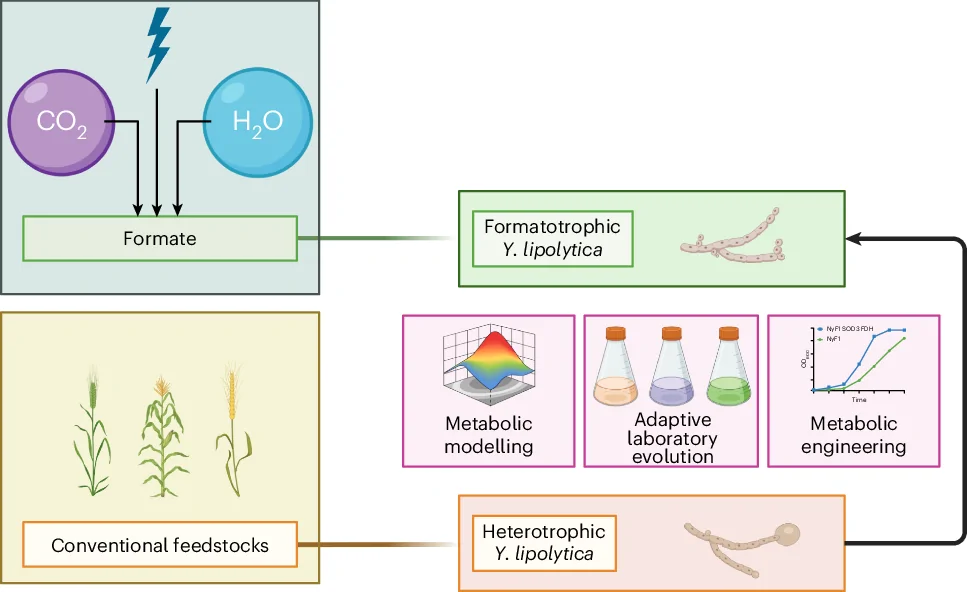

## Abstract (web summary)

*Yarrowia* *lipolytica* is a non-model yeast of considerable industrial interest. Here metabolic modelling and directed evolution are used to create a strain of *Y. lipolytica* that is able to grow on formate as a step towards electrical CO_2_-based bioprocesses based on a complex metabolic pathway.

## Main

The ongoing climate crisis demands substantial changes in the ways fuel, food and building materials are produced. Biomanufacturing is well suited to become a large part of this solution, but is constrained by the limited sustainability of conventional sugar-based feedstocks^1,2^. Formic acid can be synthesized at very high efficiency by electrically reducing CO_2_, making it an ideal sustainable feedstock for bioprocesses^3^.

Despite this potential, progress has been limited in industrializing formate-based bioprocesses. On the one hand, microorganisms with endogenous formate assimilation pathways have been explored as production hosts. However, many are difficult to engineer and have not yet shown the yields needed for industrial-scale production^4–6^. Others, such as acetogens, are strict anaerobes, limiting which products can be made^7^. Several bioproducts have been made by formate conversion via the Calvin cycle, but this is less efficient than direct formate assimilation^8–10^.

On the other hand, some headway has been made in engineering well-established industrial prokaryotes to grow on formate. *Escherichia* *coli*, *Pseudomonas* *putida* and *Cupriavidus* *necator* have all been engineered to grow on formate via the highly efficient reductive glycine pathway (RGP)^11–13^, with *E. coli* also engineered to use the serine–threonine cycle^14^. Production of lactic acid and polyhydroxybutyrate from formate was recently demonstrated in *E. coli* at relatively high yields, showing that non-native assimilation pathways can support effective bioproduction^15^. Prokaryotes, however, may suffer from scale-up issues such as susceptibility to extremes of pH^16^. Meanwhile, eukaryotes sometimes offer greater robustness and can be used to produce different compounds from prokaryotic hosts^17^.

However, eukaryotes have proved much more difficult to convert into formatotrophs, and no eukaryote is known to grow on formate as sole carbon source naturally. RuBisCo has been functionally expressed in *S. cerevisiae* and combined with a formate dehydrogenase (FDH) to supply roughly 10% of required carbon, but full formatotrophy was not observed^18^ *Komagataella* *phaffi* has been engineered to grow via the RGP^19^, however, growth is slow. *Saccharomyces* *cerevisiae* has also been evolved to grow on formate via the RGP during efforts to create a RuMP-cycle using methylotroph^20^. However, formatotrophy via other pathways has not been observed and eukaryotic formatotrophy remains poorly understood.

*Yarrowia lipolytica* is a yeast that has shown great potential for biomanufacturing^17^. Industrial-scale culture to produce a wide spectrum of organic acids and food additives has been successfully accomplished and alkane, alkene and terpenoid production has proved possible^17,21,22^. Unlike many microbes, *Y. lipolytica* is tolerant to extremes of pH, low temperatures, high concentrations of inhibitory compounds and high salt concentrations^17,23^. Furthermore, a strong endogenous flux towards lipid biosynthesis and NADPH and a high tolerance to expression burden also strengthens its bioproduction ability^24^. Therefore, engineering *Y. lipolytica* to grow on formate could provide a way to convert electricity and CO_2_ to industrial products at a much larger scale than currently possible. *Y. lipolytica* has been shown to use formate as a source of NADH during growth on glucose^25^ and formate assimilation via a cyclical assimilation pathway has been proposed on the basis of transcriptomics^26^. In this work, we use computationally guided metabolic engineering and adaptive laboratory evolution to create a formatotrophic strain of *Y. lipolytica*.

## Results and discussion

### In silico analysis of cryptic formate metabolism

**Fig. 1:**
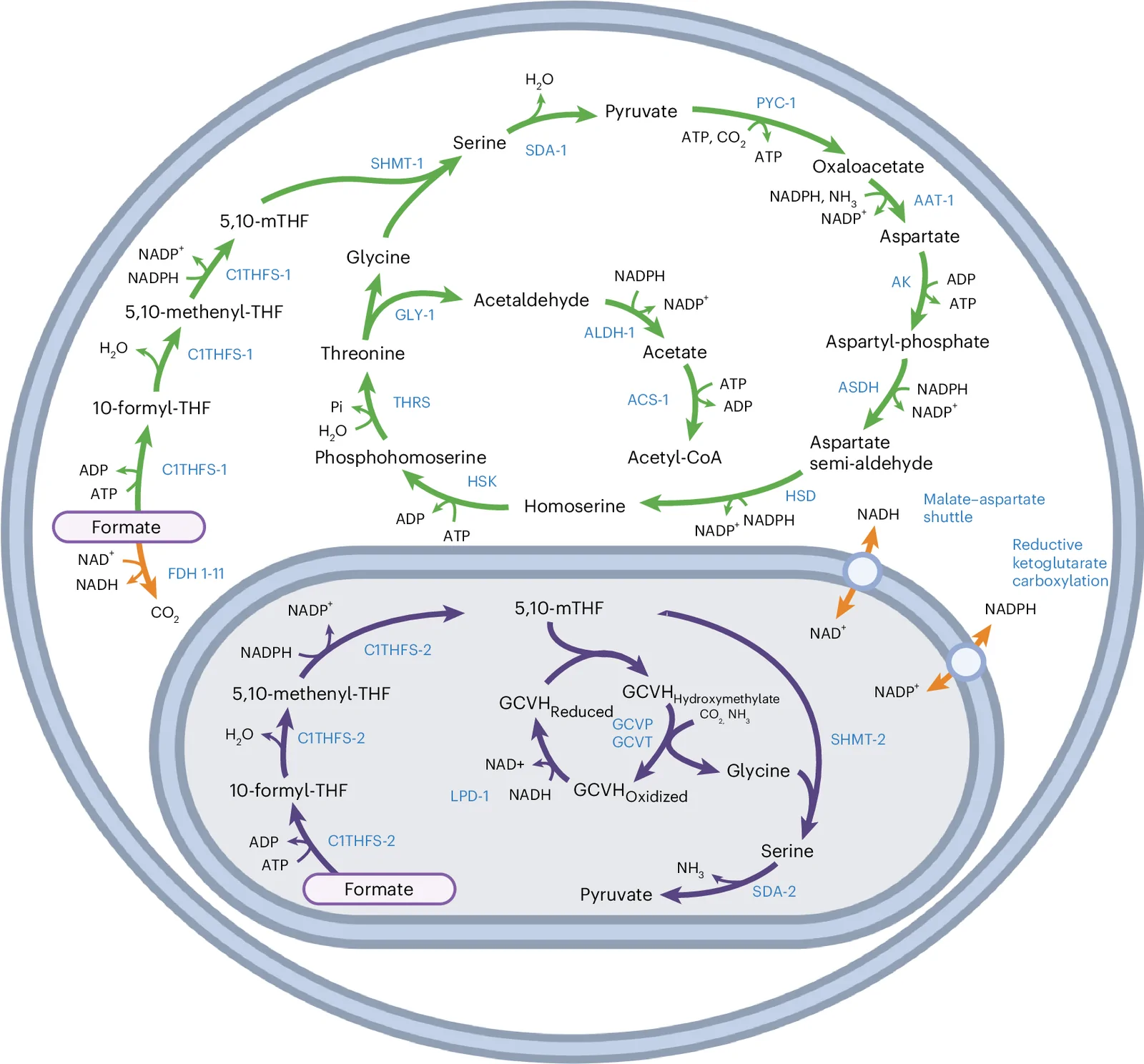
Summarized pathways of serine–threonine cycle (green) and RGP (purple). C1THFS1, tetrahydrofolate synthetase (cytoplasmic); mTHFR1, methylene-THF reductase (cytoplasmic); SHMT1, serine hydroxymethyltransferase (cytoplasmic); SDA1, serine deaminase 1; PYC1, pyruvate carboxylase 1; AAT1, aspartate aminotransferase 1; AK, aspartokinase; ASDH, aspartate semialdehyde dehydrogenase; HSD, homoserine dehydrogenase; HSK, homoserine kinase; THRS, threonine synthetase; GLY1, threonine aldolase (cytoplasmic); ALDH1, aldehyde dehydrogenase 1 (cytoplasmic); ACS1, acetate kinase 1 (cytoplasmic); C1THFS2, C1-THFS (mitochondrial), GCVL,P,T,LPD1, glycine cleavage complex; SHMT2, serine hydroxymethyltransferase (mitochondrial); SDA2, serine deminase (mitochondrial); NAD, nicotinamide adenine dinucleotide.

We initially used genome-scale metabolic modelling to find possible cryptic formate-assimilating pathways in *Y. lipolytica*. Simulating formate uptake showed that cytoplasmic methyl transfer, anapleurosis and threonine biosynthetic reactions could be patched together to assimilate formate via a cyclic pathway connecting serine and threonine (Fig. 1). Here formate is attached via 5,10-methylene-tetrahydrofolate (mTHF) to glycine to form serine. Serine is then recycled back to glycine via pyruvate and threonine. Glycine can also be synthesized using formate-derived mTHF by the RGP, via the reverse glycine cleavage system^27^. Therefore, we added this reaction to the model, which then permitted growth via the linear RGP (Fig. 1). In both cases, the model regenerates NADH and generates CO_2_ simultaneously using *Y. lipolytica*’s 11 FDHs. An analysis of the distribution of serine–threonine cycle and RGP genes using FUNGIpath showed each were widely distributed (59% and 75%) in the 166 genomes covered.

### Co-assimilation of formate during growth on glycerol

**Fig. 2:**
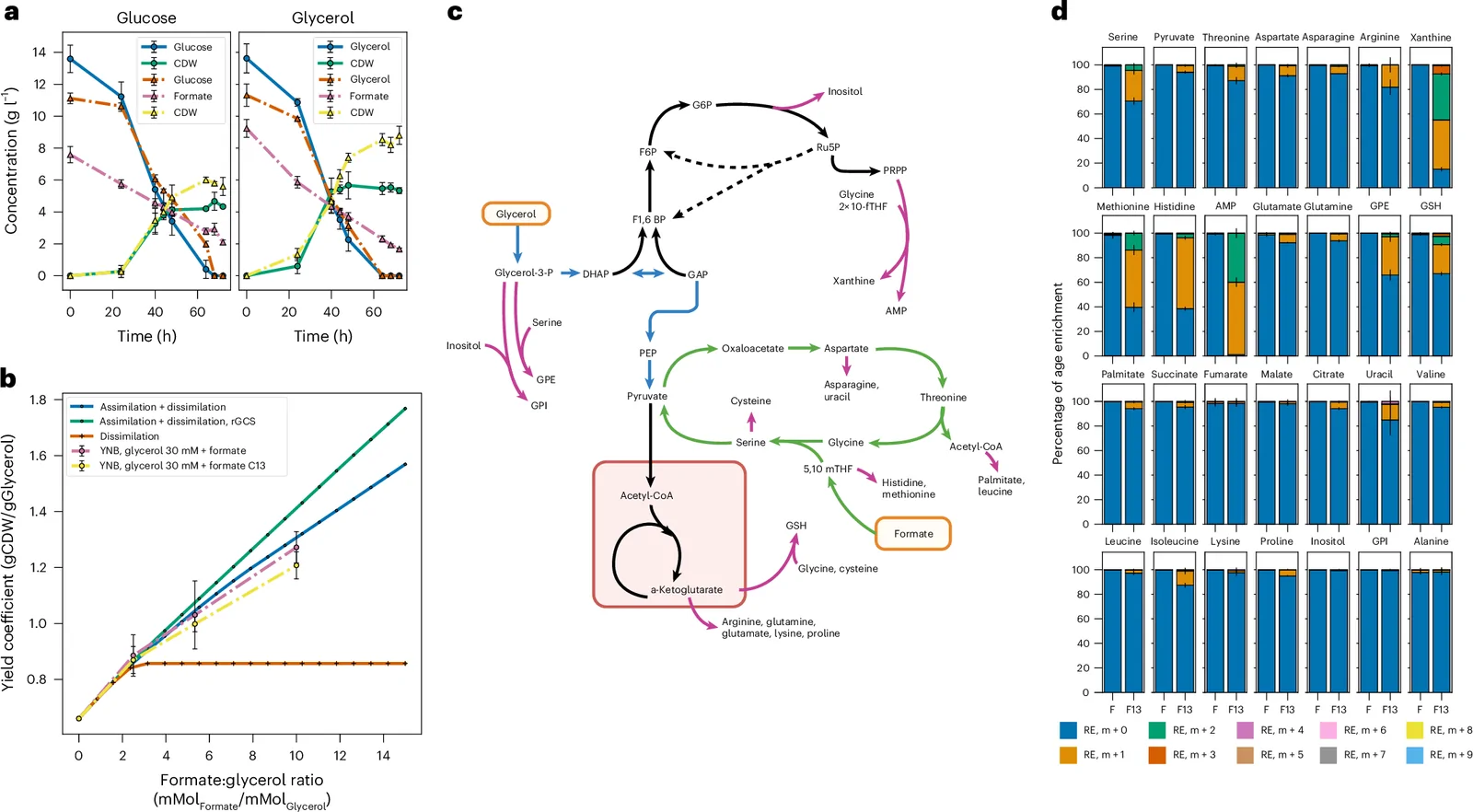
Co-consumption of formate and glycerol shows active formate assimilation. Data are presented as mean values ± standard deviation (s.d.). **a**, Consumption kinetics of formate with glucose and glycerol. The solid line indicates kinetics with carbon source only and dash-dotted line with formate added (*n* = 3, biological triplicates). **b**, Yield coefficient changes with increasing concentrations of formate added to YNB glycerol (*n* = 3, biological triplicates). Blue line: unmodified model; teal line: model with an added reversible glycine cleavage system enabling RGP activity. **c**, Schematic showing assimilation route of glycerol (blue) and formate (green). Biosynthetic routes for compounds detected by C13 tracer experiments indicated in pink. **d**, C13 tracer results of growth with glycerol and formate 150 mM (left subplot, unlabelled formate; right, labelled; *n* = 3, biological triplicates). GPE, glycerophosphoethanolamine; GPI, glycerophosphoinositol; DHAP, dihydroxyacetone phosphate; GAP, glycerone phosphate; F1,6 BP, fructose-1,6-biphosphate; F6P, fructose-6-phosphate; G-6-P, glucose-6-phosphate; Ru5P, ribulose-5-phosphate; PRPP, phosphoribosylpyrophosphate; 10 fTHF, 10-formylphosphate; 5,10-mTHF, 5,10-mTHF; RE, relative enrichment; rGCS, reversed glycine cleavage system.

Growth on formate as a sole carbon and energy source has never arisen spontaneously in any eukaryote, including *Y. lipolytica*. However, Y. *lipolytica* has been shown to consume formate under several conditions^25,26^. Therefore, we wondered whether *Y. lipolytica* host cryptic formate assimilation pathways that could be awoken with adaptive evolution. To explore formate metabolism in greater detail, we measured biomass and substrate consumption kinetics during growth on glucose and glycerol with and without formate supplementation (Fig. 2a). Cell dry weight (CDW) accumulation appeared roughly correlated with carbon source use in both cases, while formate use was roughly constant throughout growth. Biomass yield on glycerol was similar to that on glucose (Supplementary Fig. 2). However, formate addition to glycerol increased CDW yield by more than glucose: around 80% for glycerol but around 50% on glucose (Fig. 2a and Supplementary Fig. 2). We therefore decided to investigate growth on glycerol and formate in more detail. Modelling suggested that formate was used as both a supplementary carbon source via ligation to THF and a source of reducing power for NADH regeneration by FDH (Supplementary Dataset 3). These simulations showed that if formate was only used as an energy source, CDW yield plateaus at around 0.8, as growth becomes limited by the availability of carbon in the media. However, simulating use as a carbon and energy source predicts that yield can increase indefinitely if formate can be incorporated as a carbon source. When we contrasted growth via the serine–threonine cycle with the RGP, we saw that the RGP permits higher yields, probably due to the lower ATP demand. This is reflected by the higher biomass yield predicted on formate (Supplementary Fig. 1). When *Y. lipolytica* is grown on glycerol, yields match curves predicted for formate incorporation into biomass (Fig. 2b). The lower yields at the highest formate ratio for the higher glycerol concentration probably represent formate toxicity, as the concentration here is 1.5 M: around which *S. cerevisiae* also experiences impaired growth^28^. Modelling predicted that during growth via the serine–threonine cycle, formate enters metabolism via THF-mediated attachment to glycine synthesized from glycerol (Supplementary dataset 1). This forms serine, which is then converted into threonine via pyruvate carboxylation and aspartate biosynthesis. Threonine is then cleaved into glycine and acetaldehyde, which then enters the tricarboxylic acid cycle (TCA) as acetyl-CoA after conversion by acetaldehyde dehydrogenase and acetate kinase (Fig. 2c). Our analysis of C13-labelling patterns showed that serine-derived metabolites (serine, threonine, aspartate, asparagine, uracil, glycerophosphoethanolamine) were once labelled. Other mTHF-derived amino acids (histidine, methionine) were once labelled, suggesting that formate enters via the THF cycle. Adenine monophosphate (AMP) and xanthine were m + 1 and m + 2 labelled, reflecting the two molecules of mTHF used in their biosynthesis (Fig. 2c). Signs of labelled glycine were not detected: serine was only labelled once and m + 3 glutathione (GSH), AMP and xanthine, which would stem from labelled glycine, were only detected in small amounts suggesting that negligible amounts of single-labelled glycine were produced. This suggests that the RGP, which synthesizes glycine from formate, is not active (Supplementary Note 1) and instead formate enters via the serine–threonine cycle.

### mRNA sequencing shows formate-responsive transcriptional response

**Fig. 3:**
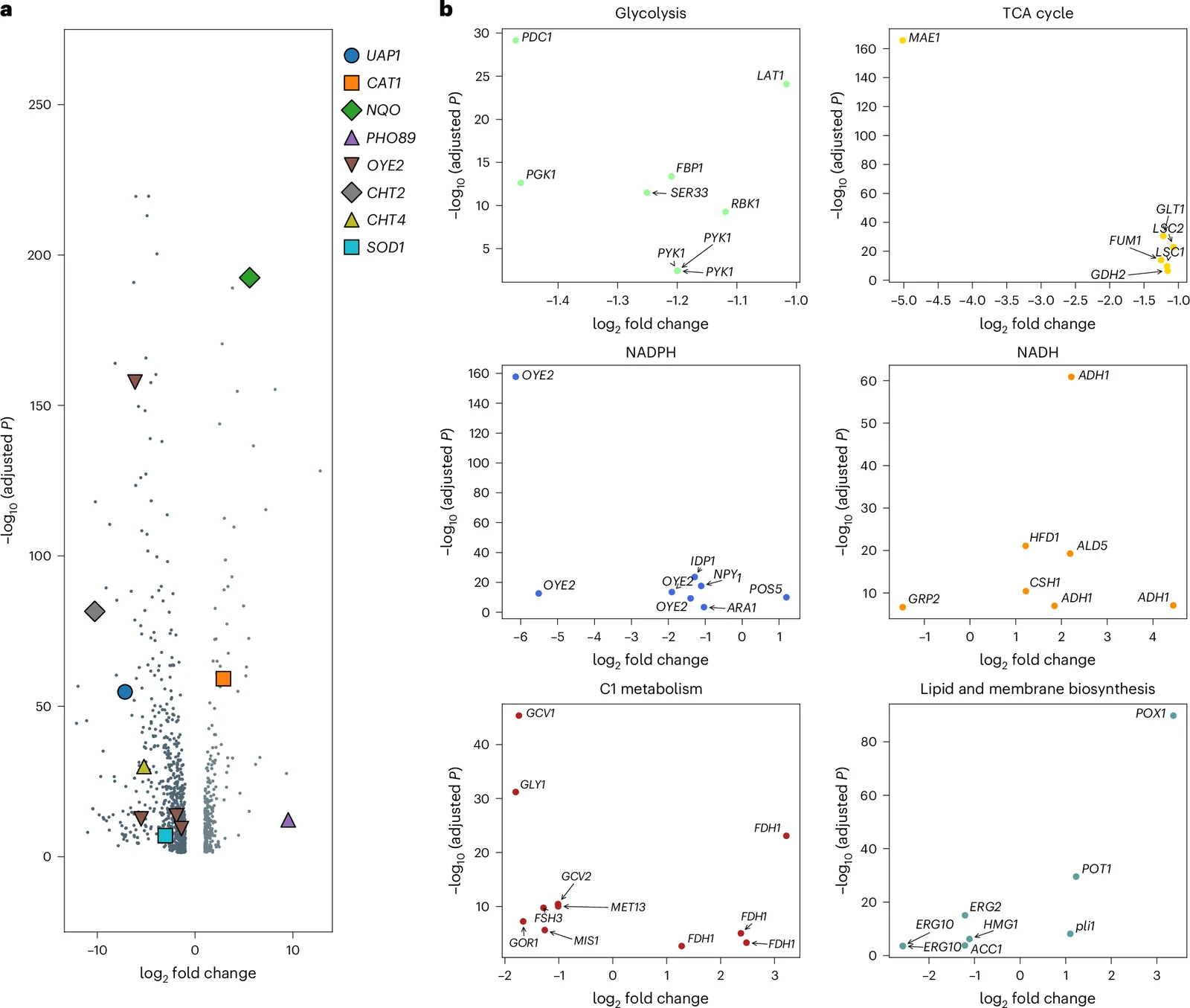
Transcriptomic changes in *Y. lipolytica* during formate exposure. **a**, Selected genes with added formate. **b**, Transcriptomic analysis of differentially expressed metabolic genes involved in major metabolic pathways: glycolysis—conversion of upper metabolism to pyruvate; TCA cycle—conversion of pyruvate to TCA intermediates and assimilation of NH_3_ via glutamate; NADPH/NADH—NAD(P)H-interconverting oxidoreductase reactions; C1—single carbon metabolism genes and lipid; and membrane biosynthesis—lipid anabolism or catabolism and mevalonate pathway genes.

**Fig. 4:**
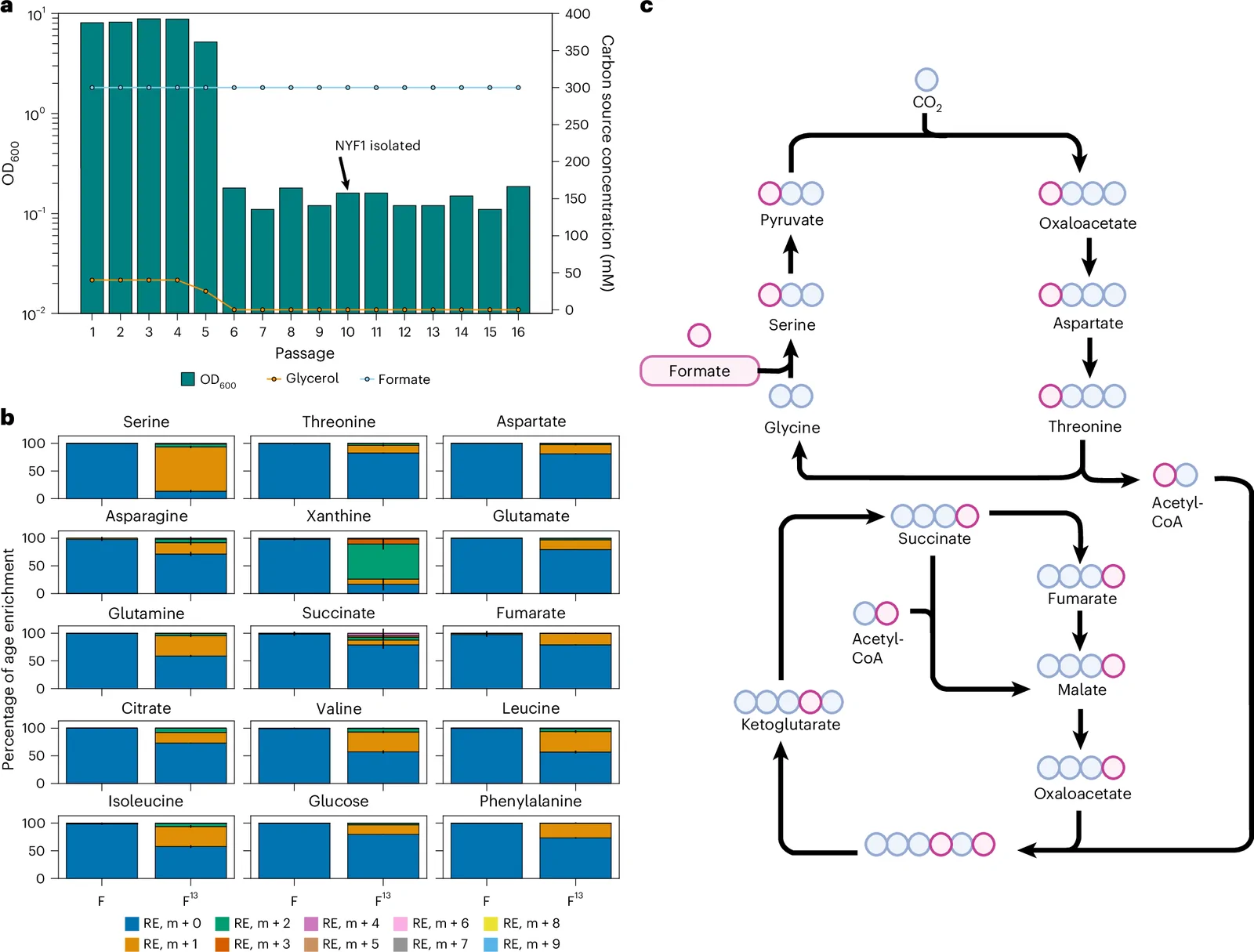
Evolution of a formatotrophic *Y. lipolytica* strain. **a**, Summarized adaptive laboratory evolution of NYF1. Teal bars indicate OD, blue and orange markers indicate formate and glycerol concentrations. **b**, C13 tracer and amino acid labelling in strain NYF1. Data are presented as mean values ± s.d. (*n* = 3, biological triplicates). **c**, Schematic showing incorporation of formate (purple) and CO_2_ (blue) into semi-labelled biomass during single passages on formate.

We next used messenger RNA (mRNA) sequencing to explore the changes in gene expression during growth on glycerol and formate. Sequencing showed widespread differences in gene expression when formate was added (Supplementary Fig. 4). As a result, we decided to focus on metabolic genes specifically, as well as focusing on highly upregulated or downregulated genes (Fig. 3). Some of these changes reflect variations in C1 metabolism. NADH is regenerated by formate oxidation via FDH, and indeed four FDH isozymes were strongly upregulated (Fig. 4b) when formate was added. We also observed strong upregulation of NQO, coding for NADH-quinone oxidoreductase. This provides another route for electrons to enter the respiratory chain and is used to restore NADH/nicotinamide adenine dinucleotide ratios under strongly reducing conditions^29–31^. TCA cycle genes coding for malic enzyme, isocitrate dehydrogenase, pyruvate dehydrogenase subunits PDC1 (E1 subunit) and Lat1 (E2 subunit) also all fell in expression level. Both TCA cycle flux and several TCA cycle genes are downregulated when *Y. lipolytica* is grown on more reducing substrates due to higher levels of NADH generated by a more reducing cellular redox status^30^. TCA downregulation could, therefore, result from NADH regeneration from FDH activity. We also observed changes in glycolysis and electron transfer genes, as well as alcohol or aldehyde dehydrogenases, which may reflect increased oxidative stress, changes in growth rate, increased acetaldehyde conversion or serine biosynthesis (Fig. 3b and Supplementary Note 2).

Formate is a known pro-oxidant as it binds and inhibits respiratory complex IV, creating radical oxygen species as electrons leak from the respiratory chain^32–34^. Several genes involved in reactive oxygen species (ROS) metabolism or resistance were also differentially expressed. Catalase, (Cat1) is a well-known marker for oxidative stress and is upregulated when formate is added. Likewise, the mitochondrial NADH kinase encoded by POS5, used to provide additional NAPDH to ROS-repair metabolism, increases in expression level. Oye2 homologues, coding for NADPH-dehydrogenases, also fall in expression level (Fig. 3a,b). Oye2 is required for hydrogen peroxide generation in *S. cerevisiae*, and so downregulation might lower oxidative stress levels^35^. Superoxide dismutase (SOD1) falls in expression, even though superoxide is frequently generated by formate toxicity^33^. Upregulation of Pho89, coding for a Na^+^/Pi efflux pump, further suggests that cells are under osmotic stress: probably arising from dissociated Na^+^ ions from the sodium formate added to growth media^36^. Overall, genes involved in cofactor biosynthesis, secondary metabolism and the electron transfer chain reduced in expression level, potentially reflecting lower growth rates^37^ (Supplementary Fig. 4b). Formate also disrupts cell membranes^38^. Consistent with this, we observed changes in the expression of lipid-related genes. *POX1* and *POT1*, genes encoding enzymes involved in lipid degradation and regulation^39^ were upregulated, while ergosterol biosynthesis genes *ERG2*, *ERG10* and *HMG1* were downregulated (Fig. 3b). Chitin biosynthesis and membrane proteins CHT2, CHT4 and UAP1 were also downregulated, reflecting altered membrane composition and potentially higher resistance to formate or osmotic stress^40^ (Fig. 3b).

### Awakening formatotrophy with adaptive laboratory evolution

We then proposed that adaptive laboratory evolution could ‘awaken’ full formatotrophy. We observed growth on formate alone after only six passages. To rule out the possibility of growth on intracellular storage metabolites such as lipids, we repeated this growth across another four passages (Fig. 4b). Growth was consistent on formate alone, with roughly 4 generations to a final optical density (OD) of 0.15 (Fig. 4b). At passage 10, strain NYF1 (Novel Yarrowia Formatotroph 1) was purified by selecting a single colony growing on formate (Fig. 4a). We tested growth of wild-type *Y. lipolytica* on formate and did not observe growth past OD = 0.05 (Supplementary Fig. 5).

C13 tracer analysis confirmed our initial modelling prediction: that growth on formate follows the serine–threonine cycle. In this case, the single-labelled serine that we observed can only arise if glycine is recycled via the serine–threonine cycle; likewise, twice labelled xanthine suggests formate assimilation via THF does not from mTHF and CO_2_ in the RGP, which would create double labelled serine and triple labelled xanthine. Similar to growth on glycerol and formate, we observe carry-through of labelled carbon to aspartate, threonine, branched chain amino acids and TCA metabolites (Fig. 4c), all of which indicate formate-derived carbon being converted to acetyl-CoA via threonine biosynthesis. Modest amounts of m + 2 labelled citrate show that 2 molecules of acetyl-CoA are assimilated via the glyoxylate cycle. This contradicts the cyclic assimilation pathway in *Y. lipolytica* proposed by one research group, where glyoxylate is predicted to regenerate glycine as opposed to being used to generate malate^26^. Single-labelled phenylalanine and glucose also demonstrate that the synthesis of gluconeogenesis metabolites also occurs from formate. *Y. lipolytica* accumulates very large amounts of lipids and carbohydrates^41^, which could support growth without formate assimilation and may dilute our C13 labelling signal. Indeed, the non-evolved control was able to sustain roughly two doublings on formate in a single passage (Supplementary Fig. 5). Therefore, we grew NYF1 on formate-only media over multiple passages. We observed consistent formatotrophic growth, and our strain failed to grow without a carbon source, making growth on intracellular storage metabolites alone unlikely (Supplementary Figs. 6 and 10).

### ROS resistance and mitochondrial mass support formatotrophy

After a simple assessment of our formatotrophic strain to investigate potential epigenetic changes (Supplementary Note 3), we decided to probe genomic and transcriptomic changes underlying formatotrophy. Whole-genome sequencing showed a small number of coding sequence mutations in transcriptional regulators and genes involved in mitochondrial morphology (Supplementary Fig. 8 and Supplementary Dataset 2). We therefore decided to use mRNA sequencing to probe transcriptional differences between wild type and NYF1. This showed a small set of changes and moderate difference (69%) between NYF1 and Po1D, with no significant enrichment of gene ontology terms (Supplementary Fig. 9).

**Fig. 5:**
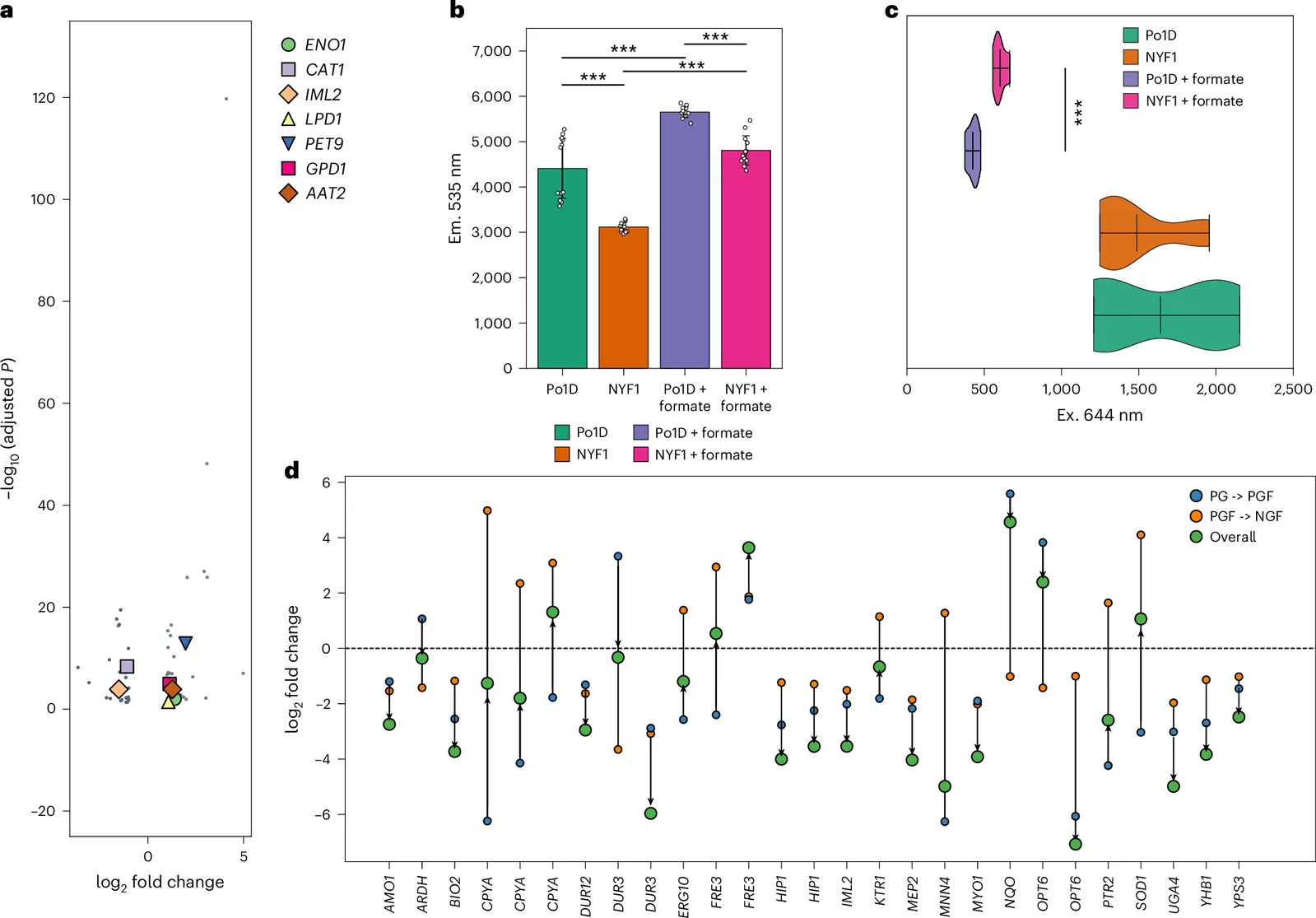
Transcriptomic and physiological changes in NYF1. **a**, Selected differentially expressed genes between NYF1 and Po1D in glycerol and formate. **b**, DCFDA assay of ROS in Po1D and NYF1 grown in glycerol with added formate (results of a two-sample paired *t*-test: *P* = 6.78 × 10^−5^, 4.55 × 10^−5^, 4.40 × 10^−10^, 6.93 × 10^−7^). Data are presented as mean values ± s.d. (*n* = 9, biological triplicates or technical triplicates). **c**, Violin plot of fluorescence at 644 nm of mitotracker-stained cells. Left and right bounds of violin shows maximum and minimum, central line indicates mean (*n* = 30, biological triplicates; *P* = 7.72 × 10^−11^). **d**, Transcript changes in differentially expressed genes shared between Po1D and NYF1. Blue marker: fold change in Po1D when formate is added. Orange marker: fold change between gene in NYF1 and Po1D. Green marker: total change from Po1D grown in glycerol to NYF1 grown in formate. Black arrow: direction of overall change; upwards: positive change; downwards: negative. Horizontal line indicates expression level of genes in Po1D grown in glycerol. All *P* values are summarized as *P > 0.05, **P > 0.01, ***P > 0.005. Ex., excitation; Em., emission.

Several metabolic genes increased in transcription level (Fig. 5a). Products of two of these, GPD1 (glycerol dehydrogenase) and ENO1 (enolase) are directly involved in glycolysis, potentially reflecting evolution towards glycerol assimilation. LPD1 also increases in transcript level. LPD1 encodes the E3 subunit for pyruvate dehydrogenase, alpha ketoglutarate dehydrogenase and the glycine cleavage system^42^. Transcription of LAT1 (encoding PDH subunit E2) falls when Po1D encounters formate (above)—increasing LPD1 transcription could counteract this and let NYF1 increase flux via PDH—a known limiting factor in growth on glycolytic substrates^37^.

We also saw that aspartate aminotransferase 1, coding for cytoplasmic aspartate aminotransferase, was upregulated (Fig. 5a). Aspartate aminotransferase interconverts oxaloacetate and aspartate, allowing electrons to be shuttled into the mitochondria via the aspartate–malate shuttle^43^. It is also required to generate aspartate in the serine–threonine cycle. Both processes should require high flux, requiring a greater amount of aminotransferase.

We also observed a clear shift towards ROS resistance, showing that, once again, formate-driven ROS production was a substantial challenge to cells. NYF1 appeared to have evolved higher resistance to ROS. For instance, expression levels of genes IML2 (encoding a DNA oxidative damage-repair protein) and Cat1 (catalase) fell suggesting lower oxidative stress (Fig. 5a). Expression levels of differentially expressed genes between Po1D and NYF1 growing in formate were also consistent with evolution towards lower levels of oxidative stress. For one, AMO1 (encoding a broad specificity amine oxidase) and various homologues of DUR3 (urea transporters) were downregulated. These participate in the allantoin degradation pathway: a product of oxidative damage of purines and a biomarker for oxidative stress^44^ (Fig. 5d). Amine oxidase may also show xanthine oxidase activity, a known source of ROS that may need to be downregulated to allow lower ROS generation^45,46^. A cytosolic superoxide dismutase (SOD1) was also increased in expression compared with Po1D. SOD1 catalyses the conversion of superoxide radicals to water^47^ (Fig. 4d), which are produced when electrons ‘leak’ from the electron transport chain during inhibition of respiratory complexes^48^ (Fig. 5a). Upregulation of SOD1 should therefore counteract radical production caused by formate inhibition of complex IV.

We then measured relative ROS production using a 2′,7′-dichlorofluorescin diacetate (DCFDA) assay and indeed found that NYF1 showed lower ROS production, both during growth on glycerol and when formate was added (Fig. 5b), suggesting that NYF1 had evolved better ROS degradation. A ROS-resistant phenotype could also improve mitochondrial function during formate exposure. Radical oxygen species promote mitochondrial depolarization and loss of function, decreasing ATP production^49^. We used Mitotracker Deep Red, a dye that binds to active mitochondria^50^, to assess whether NYF1 had more functional mitochondria. NYF1 showed a higher fluorescence at 535 nm than Po1D during growth on glycerol with formate, suggesting that mitochondria were indeed more active (Fig. 5c). Furthermore, NYF1 shows higher expression of a homologue of PET9, which encodes for a cytosolic ADP–mitochondrial ATP exchanger, which implies greater ATP synthesis^51^ (Fig. 5a). Together, these findings suggest that NYF1 has evolved greater degradation of ROS, leading to lower oxidative stress and preserving mitochondrial function when formate is added.

### Balancing NADPH and ROS metabolism improves formatotrophic growth

We then attempted to improve NYF1’s growth on formate. Extensive experiments to optimize culture conditions showed that NYF1 grew best in shake-flasks, whereas altering formate concentration, phosphate concentration, initial OD or CO_2_ availability did not change growth on formate (Supplementary Figs. 5 and 11).

We then decided to explore other strategies to improve growth. Substantial amounts of NADPH are required during growth on formate: modelling showed that almost half of all formate consumed is used to regenerate NADPH during simulated growth on formate (Supplementary Fig. 12). While several endogenous routes are available to convert electrons into NADPH, all require ATP to be spent^52^, potentially increasing the demand on oxidative phosphorylation systems already-taxed by formate poisoning. We therefore decided to express the NADPH-dependent FDH from *Burkholderia* *stabilis*^53^ (FDHbs), which converts formate into NADPH without spending ATP. This approach should also improve growth: our modelling showed that avoiding this unnecessary energy sink increases yields by around 20% (Supplementary Fig. 12).

**Fig. 6:**
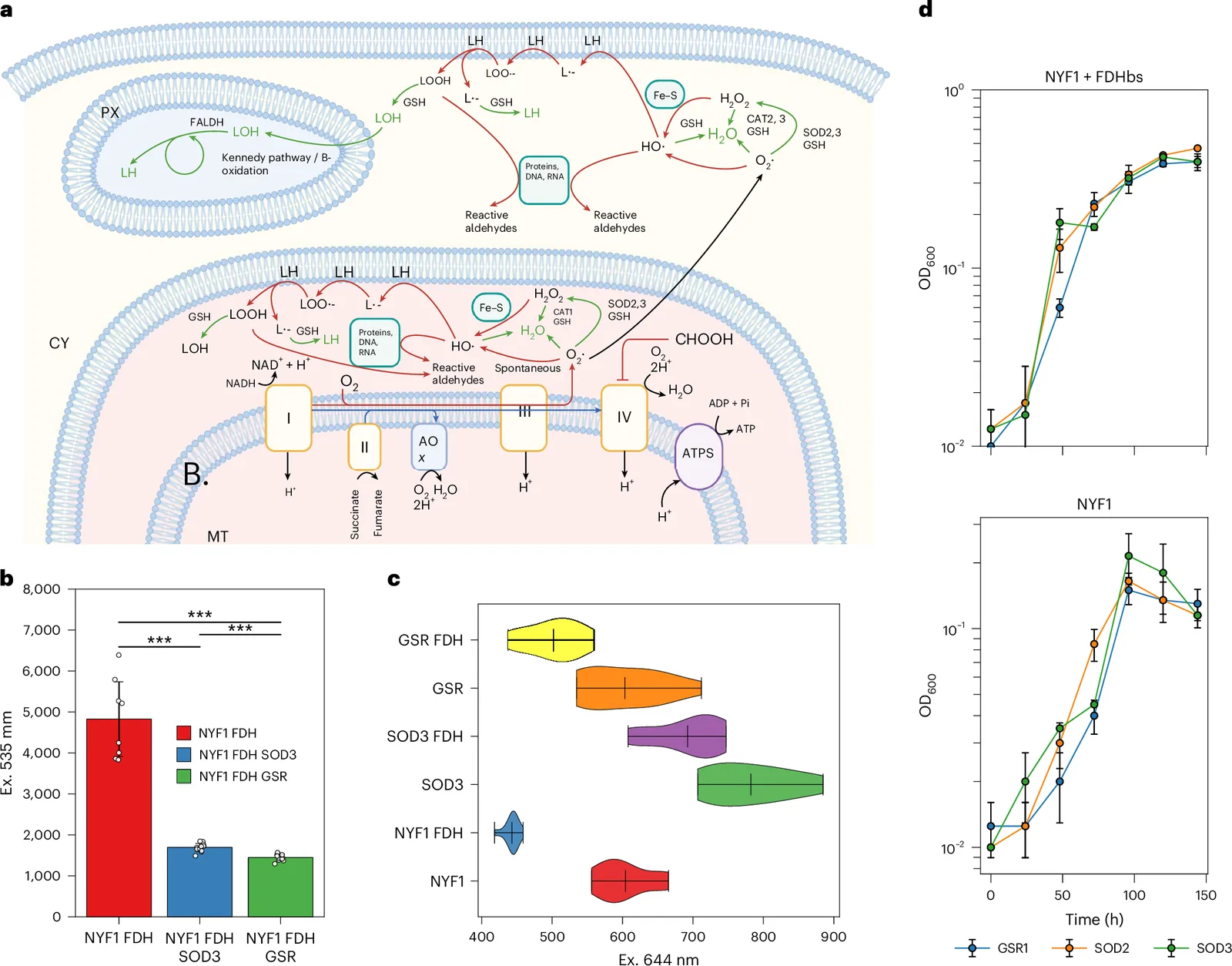
Engineering the ROS metabolism of *Y. lipolytica* improves growth on formate. Data presented as mean ± s.d. **a**, Schematic showing mechanisms of ROS-related damage in *Y. lipolytica*. MT, mitochondria; CY, cytoplasm. Blue arrow along inner mitochondrial membrane: normal flow of electrons. Red inhibitor line at complex IV: inhibitory effect of formate binding. Red arrows: flow of electrons to O_2_ to form superoxide and superoxide reactions to form hydroxonium radicals, then further reactions with lipids (LH) to form lipyl radicals and lipid peroxides (L- and LOOH). Green arrows: detoxification pathways to form water, lipid alcohols (LOH), short lipids (LH) or short aldehydes. **b**, DCFDA assay of ROS in Po1D and NYF1 grown in glycerol with added formate (*n* = 9, biological triplicates or technical triplicates; ***P> 0.001; *P*= 7.76 × 10^−11^, 1.67 × 10^−11^, 1.29 × 10^−6^). **c**, Violin plot of fluorescence at 644 nm of mitotracker-stained cells. Left and right bounds of violin shows maximum and minimum, central line indicates mean (*n* = 30, biological triplicates; *P* = 8.23 × 10^−13^, 1.62 × 10^−14^, 2.58 × 10^−5^, 4.61 × 10^−4^, 3.91 × 10^−5^). **d**, Growth curves of NYF1 or NYF1 + FDHbs expressing antioxidant enzymes (*n* = 2, biological duplicates).

Despite this, expressing FDHbs in NYF1 failed to improve growth on formate (Supplementary Fig. 13). A highly reduced NADPH pool can lead to ‘reductive stress’, where electrons leak from reduced NADPH-dependent oxidoreductases and form radical oxygen species^54^. Therefore, FDHbs expression could further increase oxidative stress, inhibiting growth. When we measured ROS production and mitochondrial activity in NYF1 + FDHbs, we observed the same trend of higher oxidative stress accompanying lower mitochondrial function as previously (Fig. 6b,c). FDHbs expression seemed to have a mild pro-oxidant effect even without formate addition, potentially the result of NADPH production from intracellular formate synthesis from the THF cycle (Supplementary Fig. 15 and Supplementary Note 4).

We then decided to test the effects of adding butylated hydroxytoluene (BHT). BHT has been used to reduce oxidation in *Y. lipolytica* cultures and is a known ROS scavenger^55,56^. BHT addition showed reduced oxidative stress in both NYF1 and FDHbs (Supplementary Fig. 15). It also resulted in better growth for NYF1 + FDHbs, with a final OD roughly double that of NYF1 (Fig. 6a). This did not occur with NYF1, suggesting that lowering ROS was required to exploit increased cellular reducing power.

Menadione stimulates superoxide formation and is known to induce ROS-tolerance in *Y. lipolytica* when added to precultures by increasing the expression of oxidative stress resistance genes^57^. Therefore, menadione addition in precultures of NYF1 + FDHbs should improve growth. Indeed, doing so showed the same pattern of better growth as seen with other antioxidant strategies (Supplementary Fig. 14).

On this basis, we experimented with overexpressing superoxide-degrading enzymes in *Y. lipolytica*. Several of these are available: superoxide dismutase 2 (SOD2) (mitochondrial) and superoxide dismutase 3 (SOD3) (cytoplasmic) catalyse the conversion of superoxide to water, while reduced GSH catalyses the degradation of a wide range of radical species, including oxygen radicals, lipid peroxyls produced by oxidative damage and hydrogen peroxide^57^ (Fig. 6a). We then expressed SOD2, 3, and the rate-limiting step for GSH production^58^ (glutathione reductase, GSR1) in NYF1. Once more, we observed better growth in NYF1 + FDHbs for all three enzymes (Fig. 6d). We then selected the two best growing strains, FDHbs + SOD3/GSR1, and measured mitochondrial function and ROS production. This showed that both strategies greatly reduced ROS production (Fig. 6b) with FDH expression. Mitochondrial function was higher when both antioxidant enzymes were expressed, with SOD3 maximizing mitochondrial output (Fig. 6c).

**Fig. 7:**
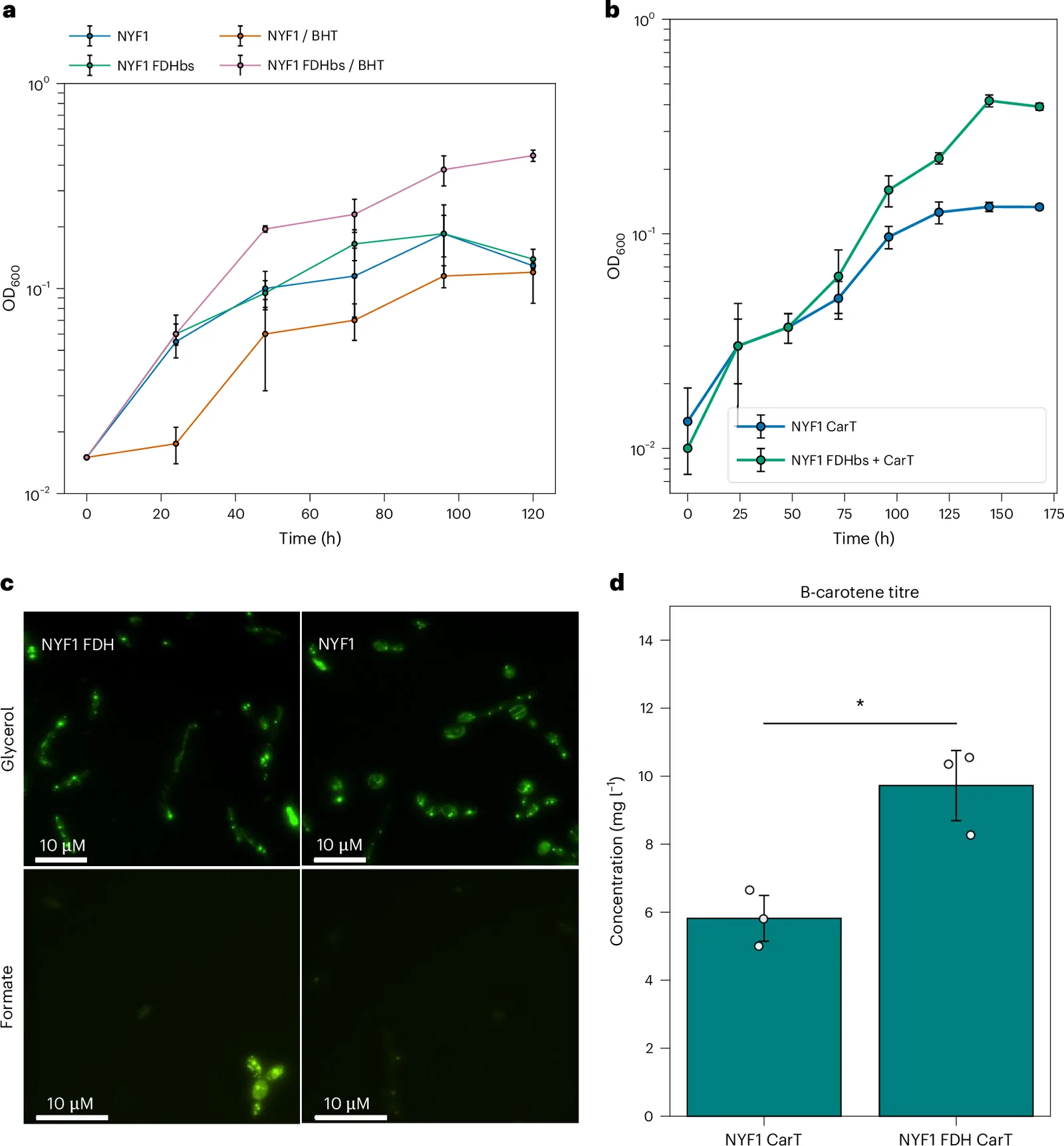
Carotene production from formate. All data are presented as mean values ± s.d. **a**, Growth curve of NYF1 and NYF1 expressing FDH from *B. stabilis* with added BHT. **b**, Growth curve of Nyf1 and NYF1 FDH expressing carotenogenesis cassette CarT. **c**, Fluorescence micrograph of NYF1 + carotenogenesis + FDH from *B. stabilis* (left) and NYF1 + carotenogenesis (right), grown on glycerol and formate (upper) or formate alone (lower). **d**, Titre and yield of carotenes produced by strain NYF1 and NYF1 with FDHbs expressing carotenogenesis genes (CarT; *P*= 0.012, *P> 0.05).

Finally, we experimented with producing beta-carotene from formate. Beta-carotene is of considerable nutraceutical interest as an antioxidant supplement, as well as being a useful benchmark for bioproduction from acetyl-CoA^59–61^. Expressing carotenogenesis genes in NYF1 allowed B-carotene production from formate (Fig. 7d and Supplementary Fig. 16). Expression of carotenogenesis genes alongside FDHbs led to a similar increase in final OD to that seen with BHT addition (Fig. 6b). Beta-carotene is known to act as a radical scavenger, which probably suppresses ROS-formation in a similar manner to BHT and so allows better growth. We then used brightfield and fluorescence microscopy to image cells during growth on a relaxing carbon source (glycerol) and during formatotrophic growth (Fig. 6c). This showed that beta-carotene was localized to lipid bodies commonly seen with beta-carotene in *Y. lipolytica*^62^. Production was high overall, with a high proportion of cell mass made up of accumulated carotenes (Supplementary Fig. 16). Carotene production per cell was lower in the better growing FDHbs strain, suggesting that there may be a trade-off between carotene and biomass production.

While our modelling shows that a biomass yield of around 6 g l^−1^ is possible with the serine–threonine cycle, our strain achieved lower CDW concentrations, around 0.6 g l^−1^ (Supplementary Fig. 1). This reflects the difficulty encountered when engineering C1-trophic metabolism. For example, when one research group replaced the Calvin cycle with the RGP, their initial formatotrophic strain only achieved around 50% of the theoretical maximum due to non-optimized gene expression and unexpected metabolic side reactions^12^. Therefore, a long road lies ahead, with further engineering required to balance the complex network supporting formatotrophic metabolism

## Conclusions and perspective

*Y. lipolytica* is a yeast with considerable industrial applications, and here we have evolved it to grow on formate. We observed growth via the eukaryotic serine–threonine cycle and noted that formatotrophy arose via evolution alone. In two other examples of eukaryotic formatotrophy, poor energy generation was suggested to be a limiting factor of growth on formate^19,20^. In this case, *Y. lipolytica*’s ability to increase the expression of several FDH genes probably allows it to generate large amounts of NADH to sustain energy-hungry formate assimilation, making it a strong prospective platform for C1 bioprocesses.

Moreover, *Y. lipolytica* appears to be a cryptic formatotroph; no genes involved in formate assimilation were upregulated after formatotrophy evolved and growth on formate and glycerol was highly efficient, implying that existing metabolism could support formate incorporation. ROS degradation is consistently involved with supporting growth on formate. Given that formate is common in the environment^63^ and a potent respiratory chain inhibitor^38^, this cryptic metabolism could represent a detoxification mechanism that allows *Y. lipolytica* to avoid poisoning by assimilating environmental formate to biomass. Finally, we show that metabolic engineering of cofactor metabolism and oxidative stress resulted in a twofold higher final OD, as well as allowing strong carotene biosynthesis. Efforts to increase cellular reducing power—important during growth on oxidized carbon sources—appeared to generate further oxidative stress. This seemed to counteract the benefits of better cofactor regeneration unless ROS production was countered by improved antioxidant metabolism. With these findings in place, future research efforts should focus on improving ATP generation, ROS resistance and maximizing flux via the serine–threonine pathway. Experiments further optimizing physico-chemical culture parameters, such as pH, oxygenation and carbon source feeding could also be explored as an avenue to improve growth. This work demonstrates the capacity for complex cryptic metabolism to emerge with surprising speed. The eukaryotic serine–threonine pathway is widespread in fungi and could therefore support formatotrophy in a wide range of organisms, furthering the development of a fungal C1-trophic bioeconomy.

## Methods

### General strain engineering

*Y. Lipolytica* engineering was carried out according to protocols developed in refs. ^64^ and ^65^. Overexpression constructs were constructed with Golden Gate assembly of Tef, Tef4UAS or Tef8UAS synthetic promoters, the gene of interest, a LipT1 terminator and an integration vector carrying a nourseothricin (pZNS-1.3/pJMP62-NAT) or hygromycin resistance marker (pJMP62-Hyg), and Zeta transposon-mediated random integration sites (sequences in Supplementary Tables 2 and 3). Endogenous genes of interest were amplified by PCR from purified *Y. Lipolytica* chromosomal DNA with primers bearing appropriate overhangs with Q5 polymerase and inserted by restriction ligation into expression vector pZNS1.3. Codon-optimized FDH (GenScript) from *B. stabilis* was expressed under promoter pTEF with a Noursethricin marker from the JMP series of zeta-mediated integration vectors (sequences in Supplementary Table 2)^66^. All *Y. lipolytica* transformations were carried out via a standard chemical method using 2 M dithiothreitol and lithium acetate^67^. Following transformation, cells were plated on either rich media (yeast extract + 2% glucose and 1% sodium formate) with 250 mg ml^−1^ nourseothricin or defined minimal media (yeast nitrogen base (YNB) + 2% glucose, 2% citrate, 1% sodium formate and 100% synthetic complete uracil dropout supplement). Glycerol stocks were made up to 25% glycerol by mass and the appropriate volume of overnight culture made in selective media. Colony PCR was carried out using primers binding to the gene of interest and the Tef promoter of synthetic constructs (sequences in Supplementary Table 4). Laboratory *Y. lipolytica* strain Po1D with restored prototrophy was used as the base strain for high-performance liquid chromatography (HPLC) analysis and initial evolution experiments, as well as controls for strain engineering studies. The formatotrophic strain, NYF1, was isolated by streaking out the mixed culture on rich media followed by growth characterization of a single colony.

### Genome-scale metabolic modelling of yield coefficients

Genome-scale metabolic modelling was carried out using the COBRA toolbox for Python and the *Y. lipolytica* genome-scale metabolic model, iYali4.2, developed in ref. ^68^. Theoretical yield calculations were accomplished by setting formic acid uptake rates to 1 mmol gCDW^−1^ h^−1^ and running Flux Balance Analysis as included with the base COBRA toolbox. New reactions corresponding to heterologous pathways were added by manually inserting reactions with appropriate reaction stoichiometry to the model. Expected yields for co-use modelling were calculated by setting carbon source uptake rates to 1 g per gram of biomass. Formic acid was either fed to the model as a supplementary carbon source using the formate metabolite provided with iYALI, or as a pseudometabolite only able to regenerate one molecule of NADH. Resultant yields were divided by the molar mass of each carbon source to derive yield coefficients. Any OD–CDW conversions required were assumed to follow the correlation reported in ref. ^69^ (CDW = 1.26 × OD − 0.35).

### Microorganism cultivation for growth assays

*Y. lipolytica* strain Po1D (uracil and leucine auxtroph bearing restored prototrophies) was used as the base, ‘wild-type’ strain. Growth experiments were carried out in minimal media (YNB) with the stated carbon source concentration, with 50 mM sodium diphosphate buffer added to maintain neutral pH. C/N ratios for growth characterization experiments were maintained at 20/1 by varying ammonia concentration in the media. Formatotrophic strains were grown at ‘high’ ammonia concentrations (60 mM, C/N = 5). Strains were grown on formate as a sole carbon source at 300 mM sodium formate (Sigma). Culture conditions were varied between narrow, wide-necked and baffled flasks purchased from VWR, Corning 98-well or 24-well plates placed in an Infors HT shaking incubator set at 250 rpm. All experiments were carried out in duplicate (Figs. 5d and 6a and Supplementary Figs. 5–7, 13 and 14) or triplicate (Fig. 6d) and statistical significance was assessed using a two-tailed Student’s *t*-test. Timepoint OD_600_ readings were taken with a Synergy HTX plate reader from Agilent Technologies. Experiments at high-CO_2_ concentrations were carried out in 50-ml flasks shaken at 250 rpm in an Algaetron AG230 incubator with CO_2_ maintained at 10,000 ppm (1%). All OD_600_ readings were normalized to spectrophotometer readings by multiplying by the difference between spectrophotometer and plate reader path length (10:1). Blank measurements were made using non-inoculated media for each growth experiment. Precultures were made 24 h before experiments in yeast extract peptone dextrose 0.5% glycerol + carbon source of interest, followed by a day culture of 6 h before inoculation of each growth experiment.

### C13-labelling experiments

For C13-labelled intracellular analysis experiments, strains were cultured in 20 ml of media in 50-ml flasks. For labelling experiments, YNB + 30 mM glycerol + 150 mM formate was used. For C13 labelling of the formatotrophic strain, cultures were grown in YNB + 300 mM formate in triplicate.

For tracer experiments, metabolic reactions were quenched by adding 20 ml of precooled (−20 °C) methanol to 15 ml of culture in an ethanol-dry ice bath, followed by vortexing. Cells were washed by centrifugation at −10 °C and 4,000*g* and resuspension in methanol, and pellets were then dissolved in 2.5 ml of 75% ethanol at 85 °C. After incubation for 5 min, suspensions were vortexed to completely lyse cells and frozen by immersion in dry ice. Debris was pelleted by centrifugation and 1 ml of supernatant was recovered for liquid chromatography–mass spectrometry (LC–MS). For amino acid labelling in the formatotrophic strain, cell pellets left over from tracer experiments were resuspended in 4 ml of 6 M HCl and incubated at 75 °C overnight to lyse proteins. Lysates were dried by incubation at 75 °C for a further 24 h in open Falcon tubes. Amino acids were resuspended in 500 μl of H_2_O, spun to remove insoluble cell debris and used as analyte. Labelled metabolites were detected using an Agilent Infinity ultra-performance liquid chromatography system and 6550 quadrupole time of flight mass spectrometers. Metabolites were separated with an Agilent Zorbax Eclipse Plus C18 column (2.1 × 50 mm, 1.8 μm) at 0.3 ml min^−1^ flow rate. Solutions of 0.1% aqueous formic acid (inorganic) and 0.1% formic acid in acetonitrile (organic) were used as mobile phase. Mobile phase gradient was moved from 2% to 98% organic over 2.5 min, followed by 1 min of constant concentration. An The Agilent JST electrospray interface in negative ion mode was used to generate spectra.

C13 relative enrichment values were calculated by first calculating relative percentages from corrected ion percentages. These were found by dividing m + 1 corrected ion percentages by m + 0 percentages. Relative enrichment values were then calculated by subtracting average control corrected ion percentages from labelled replicates and dividing by the total relative percentage. In the case of formate-only growth, corrected ion percentages were corrected for unlabelled biomass contamination by dividing each m + 1, 2 (and so on) value by the OD reached by Po1D on formate over the average final OD of NYF1 grown on formate. Statistical significance was assessed using a two-tailed Student’s *t*-test.

### HPLC and biomass yield analysis

HPLC experiments were carried out using a Thermo Scientific U3000 Standard HPLC machine. Mobile phase for metabolite analysis was composed of 5 mM H_2_SO_4_ at 0.6 ml min^−1^ and an Aminex HPX-87H column for sugar detection set at 30 °C was used for chromatography of hydrophilic metabolites. Flow rate was set at 0.6 ml min^−1^, with ultraviolet detection at 210 nm. Standards of each metabolite of interest were prepared by making up volumetric flasks at 20 g l^−1^ and creating dilution series from 0.1 g l^−1^ to 20 g l^−1^. Calibration curves resulting from this were then used to detect metabolite concentrations using Chromeleon v.6.8 software. Samples were prepared by taking 250 μl of culture, centrifuging at 14,000*g* for 5 min and transferring supernatant to HPLC vials. Volumes were then made up to 500 μl with 250 μl of Millipore-purified water. For initial consumption experiments, 250-ml flasks were used. Evaporation from these flasks was accounted for by assuming 25% volume evaporation per day. In initial yield experiments, glycerol and glucose concentrations were normalized to 2% glucose by dividing enthalpies of formation: formate was supplemented at 20 g l^−1^.

Dry biomass readings were made as follows: 2 ml of washed culture was transferred to weighed and predried 2-ml microcentrifuge tubes, and then dried for 72 h. Masses were then recorded using an Ohaus Pioneer PX142M analytical balance. Final biomass was calculated by subtracting the residual carbon in the media from the raw mass data. All experiments were carried out in triplicate.

### ROS-response characterization experiments

Strains were cultivated as stated previously for experiments with formate. Where stated, 0.01 mM menadione was added to precultures for 6 h before growth experiments started. Alternatively, 0.01 g l^−1^ BHT was added to growth media by making a 10 g l^−1^ BHT stock solution in 100% acetone and adding as needed. BHT flasks were then vortexed to prevent precipitation of the hydrophobic antioxidant. ROS quantification was carried out using a DCFDA assay. Briefly, cells were grown at 30 °C on glycerol or glycerol + formate, then collected by centrifugation and washing in PBS. Cells were stained with 100 μl of 20 μM DCFDA solution, incubated for 30 min at 30 °C, then measured for fluorescence at excitation and emission of 485 nm and 535 nm, respectively, in an Agilent Synergy H1 plate reader. OD_600_ was also assessed to normalize data. Measurements were carried out three times, in biological triplicates. Statistical significance was assessed using a two-tailed Student’s *t*-test.

### Carotenoid bioproduction

Carotenoid producing strains were constructed using a hygromycin-resistant 3 gene overexpression cassette based on JMP vectors with GGS1, CarB and CarRP under strong synthetic Tef8UAS promoters^70^. Carotenoid producing strains were streaked to single colonies, with the strongest orange colony picked to ensure strong carotenoid production. Before culture in formate, carotenoid producing strains were cultivated in yeast extract peptone dextrose glycerol + 300 mM formate for 96 h to allow carotenoid accumulation, followed by the standard 6-h day culture before transfer to growth experiment media. Carotenoid expressing strains were cultured in 40 ml in 250-ml flasks of YNB formate 300 mM. Carotenoids were extracted for HPLC by spinning 40 ml of culture volume down, resuspending in 1 ml and then bead-beating in 100% acetone at 6,037*g* for 60 s. Tubes were then centrifuged at 4,193*g* for 3 min and supernatant removed for analysis. Carotenoids were detected by HPLC using a YMC Carotenoid C30 column at 455 nm. All growth and production experiments were carried out in triplicate. Micrograph images of carotenoid producing cells were taken in fluorescence and brightfield mode by placing 10 μl of culture media directly on slides. Culture media was either concentrated 100× by centrifuging (formate-only media) or taken directly from flasks. Statistical significance was assessed using a two-tailed Student’s *t*-test.

### mRNA and DNA sequencing

Samples for mRNA sequencing were prepared by growing cells in flasks with 20 ml of YNB + 30 mM glycerol (Po1D) or glycerol + 150 mM formate (Po1D, NYF1). Experiments were carried out in triplicate. Cells were collected at the end of exponential phase, pelleted by centrifugation at 4,193*g* at −10 °C and immediately frozen on dry ice. Whole cell pellets were shipped for commercial mRNA sequencing by Azenta Life Sciences Ltd. Briefly, mRNA fragmentation and random priming were used to append poly A tails to RNA, followed by complementary DNA amplification. dA tailing and 5′ phosphorylation was then used to prepare fragments for adaptor ligation, after which PCR enrichment was used to amplify strands for sequencing. Reads trimmed using Trimmomatic v.0.36 and mapped to *Y. lipolytica* genome assembly ASM3722351v1 using a splice alignment algorithm (STAR aligner v.2.5.2b). Unique mRNA reads were calculated with Subread v.1.5.2, with reads outside intron regions discarded. DESeq2 was then used to generate *P* values and log_2_ fold values using the Wald test for pairwise differences. Adjusted *P* values >0.05 and log_2_ fold change >1 were called as differentially expressed genes for analysis and repeated reads were removed. Upregulated genes were functionally annotated by homology to known sequences in the EggNOG database using eMapper with gene names reported as the ‘preferred names’ output for ease of interpretation. Functional clustering of genes for both strain Po1D and NYF1 was carried out using PantherDB to assess clustering in gene ontology terms for molecular functions, biological processes or cellular components. Clustering was carried out with Fisher’s exact test with Bonferroni correction for multiple testing, with a *P* < 0.05 cut off. All mRNA sequencing experiments were carried out in triplicate.

For assessment of metabolic genes, entries were sorted by annotation (above) into TCA cycle, glycolysis, NADH, NADPH, cofactor, C1, lipid and membrane, electron transport chain or secondary metabolism-related genes on the basis of their annotated reactions. Here C1 metabolic genes were genes involved in C1-related reactions or assimilation pathways; NADH/NADPH were genes involved in NAD(P)H dependent redox reactions; lipid and membrane biosynthesis genes were biosynthetic genes for lipids or cell membrane components; electron transport chains were specific electron transport chain components; cofactor metabolic genes as genes involved in cofactor biosynthesis; TCA or glycolysis genes as nodes in the TCA cycle or glycolysis or gluconeogenesis (including serine biosynthesis via 3PG) and secondary metabolism genes taken to be all other genes involved in metabolite biosynthesis (amino acids, sugars and so on).

NGS DNA sequencing of strain Po1D and NYF1 was carried out by Biomarker Technologies GmbH. Whole biomass samples of each were prepared as above with strains grown in rich media for 48 h. Reference genome GCA_000002525 (strain CLIB 122) was used, with 90% of NYF1 genes and 88% of Po1D genes correctly mapped. S1000 Analysis Pipeline was used to assemble genomes using in-house methods. Single-nucleotide polymorphisms and small insertion–deletion (InDel) mutations were detected using the HaplotypeCaller algorithm from GATK software to detect variations between clean reads and the reference genome. Twenty-four single-nucleotide polymorphisms and three small InDels not present in Po1D were found in NYF1. Of these, three single-nucleotide polymorphisms and two InDels appeared in coding regions of genes. DIAMOND was used to functionally annotate genes of interest using NCBI’s Non Redundant database (NR), SwissProt, Gene Ontology terms, Clusters of Orthologous Gene (COG) terms and Kyoto Encyclopedia of Genes and Genomes terms. Assessment of distribution of serine–threonine cycle or RGP genes was carried out using FUNGIpath’s pathway explorer tool^71^. A custom BioPax2 script was used to search for EC numbers belonging to genes from each pathway across 166 fungal genomes (Supplementary Dataset 1). Genomes lacking one or more genes were assumed to be incapable of supporting the given pathway.

### Mitochondrial mass quantification and mitochondrial imaging

Comparative mitochondrial mass was measured using Mitotracker Red FM. Here 1 mM stock solutions were made up by dissolving 50 μg in 92 μl of dimethyl sulfoxide, followed by dilution to 200 nM by adding 0.4 μl to 2 ml of PBS. Strains Po1D and NYF1 were grown in 2% glycerol or 2% glycerol + 300 mM formate in duplicate, then added at an OD_600_ of 1.0 into 100 μl of 200 nM Mitotracker Red FM. Cells were stained by incubation at 30 °C for 30 min, then spun at 10,733*g* for 10 min to pellet cells. Cell pellets was resuspended in fresh PBS and measured for fluorescence and OD_600_ in a Synergy H1 plate reader in triplicate (excitation 581, emission 644 nm) 10 times. Mitochondria were imaged using fluorescence (excitation 581, emission 644 nm) and brightfield microscopy after 30 min of staining with 200 nM Mitotracker Red FM in PBS. Statistical significance was assessed using a two-tailed Student’s *t*-test.

### Reporting summary

Further information on research design is available in the Nature Portfolio Reporting Summary linked to this article.

## Supplementary information

Supplementary Information Supplementary Figs. 1–16, Notes 1–4 and Tables 1–4.

Reporting Summary

Supplementary Data 1 Supplementary Dataset 1.

Supplementary Data 2 Supplementary Dataset 2.

Supplementary Data 3 Supplementary Dataset 3.

## Data Availability

Data for sequencing, genome-scale metabolic models and analysis of pathway distribution is included in Supplementary Datasets 1–3. Sequencing data can be found at the NCBI BioSample database under accession numbers SAMN50287922, SAMN50287923, SAMN50287924, SAMN50287925, SAMN50287926, SAMN50287927, SAMN50287928, SAMN50287929, SAMN50686001 and SAMN50287930. Data supporting the findings of this study are available via Figshare at https://doi.org/10.6084/m9.figshare.31249870 (ref. ^72^).

## Code availability

Code for all modelling is available via GitHub at https://github.com/WNewell21/LatentC1YLip25.
