# Supplementary material for: Engineering de novo formatotrophy in the non-model yeast Y. lipolytica
Source: Nat Synth. 2026 Jun 12;5(9):1360–73. doi: 10.1038/s44160-026-01076-7 (PMC13553303; doi:10.1038/s44160-026-01076-7)
Supplement: Supplementary file 1 [file 44160_2026_1076_MOESM1_ESM.pdf]

nature synthesis

Supplementary information https://doi.org/10.1038/s44160-026-01076-7

Engineering de novo formatotrophy in the non-model yeast *Y. lipolytica*

In the format provided by the authors and unedited

Supplementary materials

Contents

Supplementary materials....1
Figure S1....2
Figure S2....2
Figure S3....3
Figure S4....3
Figure S5....4
Figure S6....4
Figure S7....5
Figure S8....5
Figure S9....6
Figure S10....6
Figure S11....7
Figure S12....7
Figure S13....8
Figure S14....8
Figure S15....9
Figure S16....10
Supplementary Note 1....10
Supplementary Note 2....11
Supplementary Note 3....12
Supplementary Note 4....12
Strains used in this study....13
Gene sequences of overexpression constructs....13

Figure S1

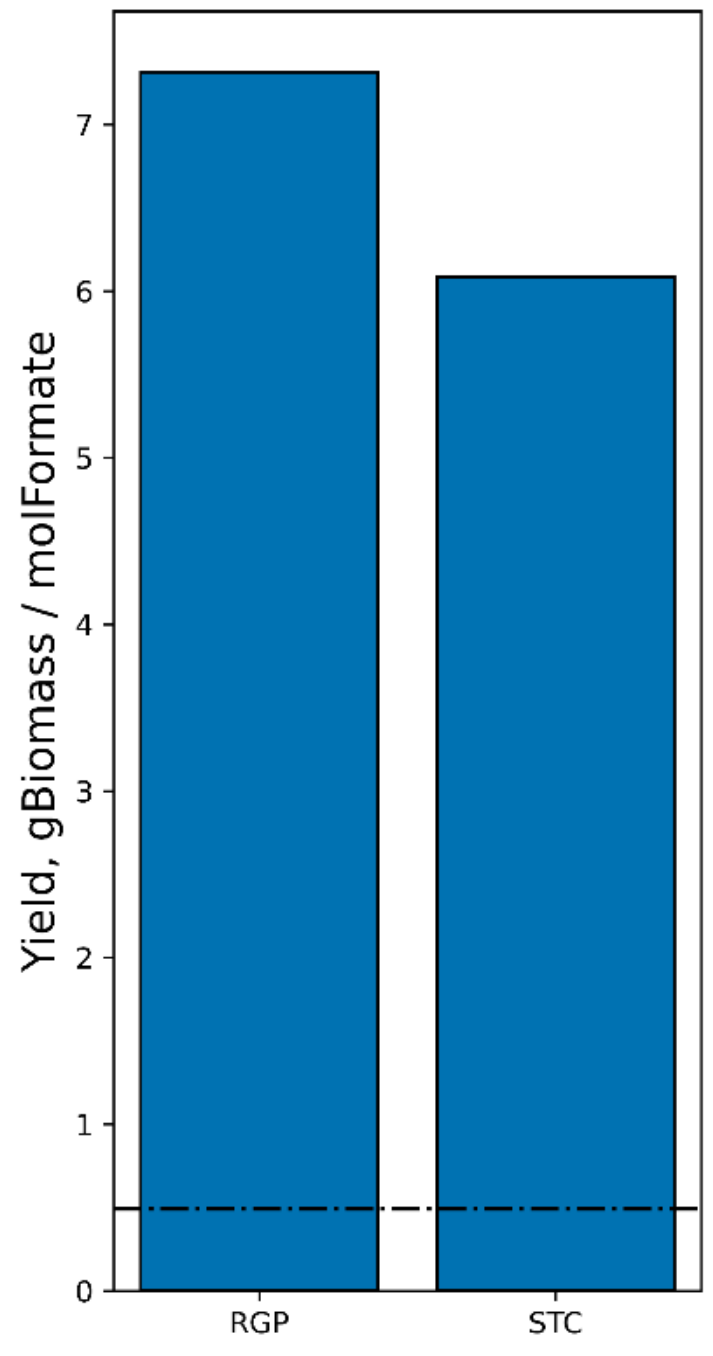

Fig S1: Predicted yields on formate via reductive glycine pathway (RGP) and serine-threonine cycle (STC). Actual yield (0.56 gCDW / mFormate) of strain NYF1 SOD3 FDHbs as dotted line.

Figure S2

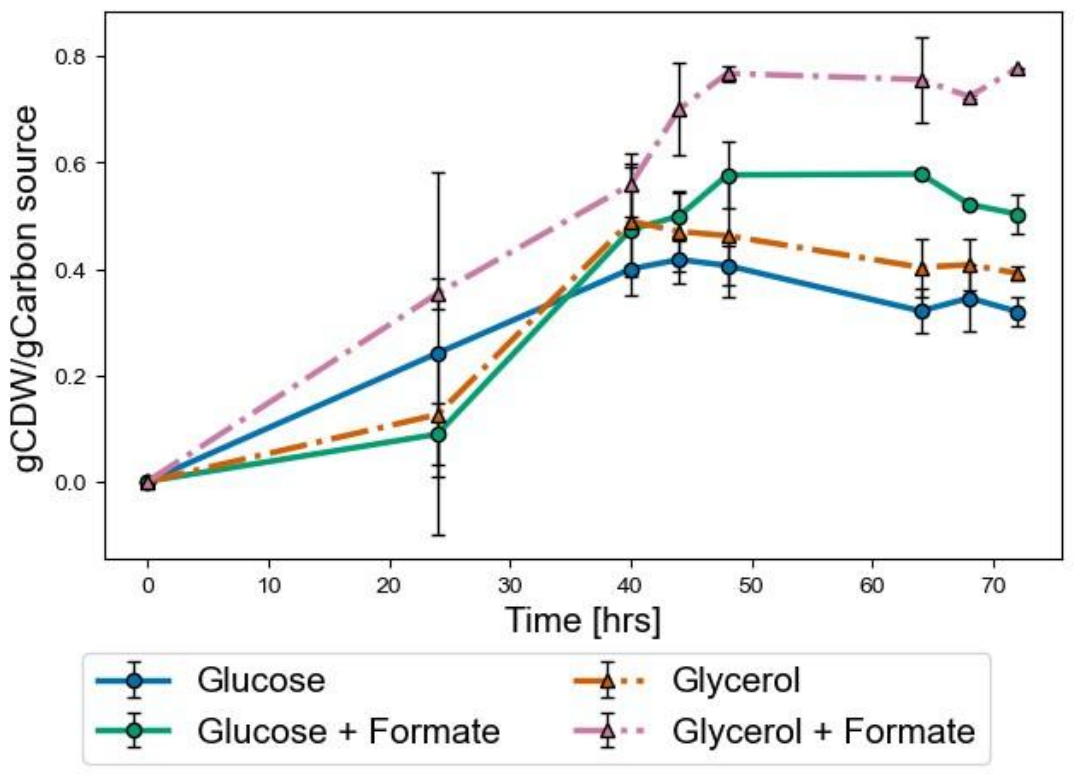

Figure S2: Cell dry weight changes over time during growth in glucose (100mM) or glycerol (90mM). N=3, biological triplicates.

Figure S3

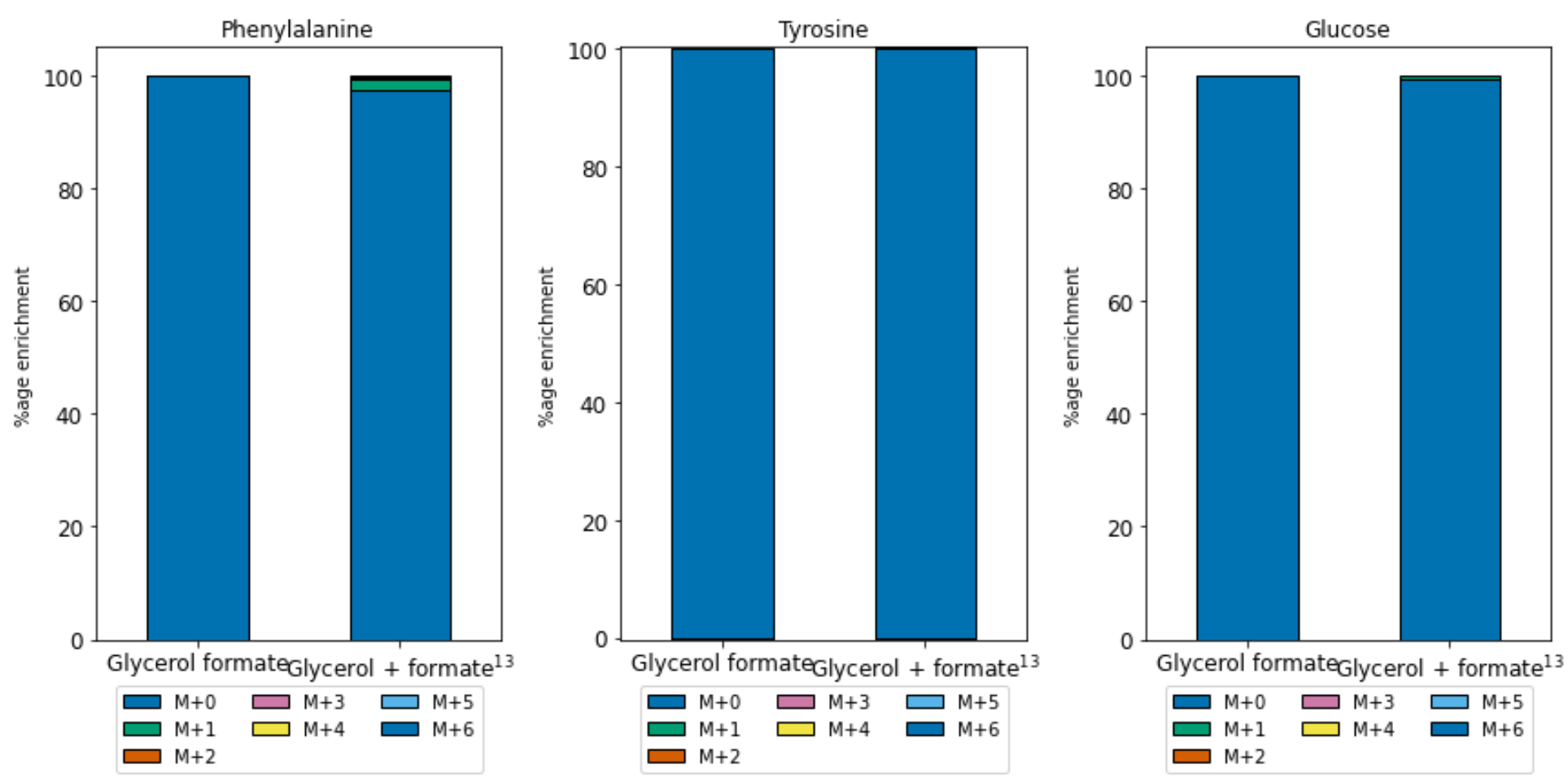

Figure S3: C13 tracer data for phenylalanine, glycose and tyrosine labelling during growth on glycerol + formate. Data are presented as means +/- SD, n=3, biological triplicates.

Figure S4

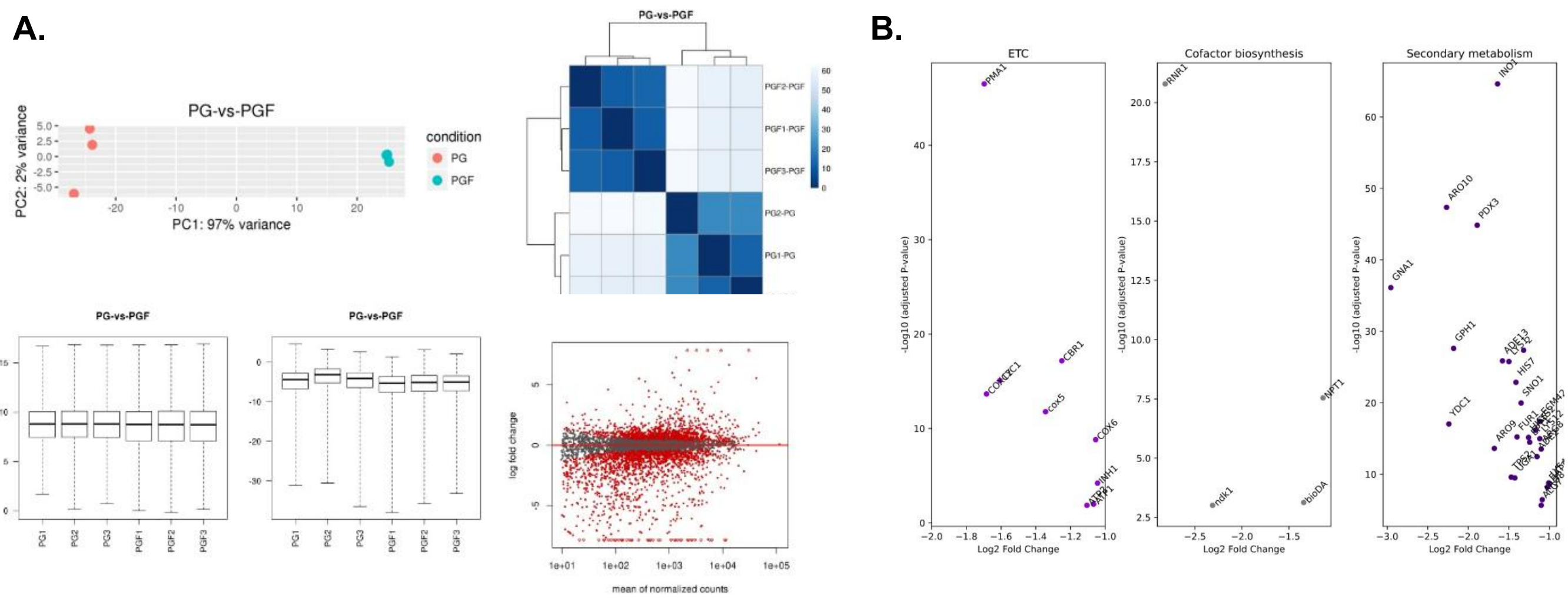

Figure S4: A: PCA of Po1D grown in glycerol and glycerol / formate (upper left). Lower left: box plot of normalised (left) and raw (right) average gene expression values. Upper right: heatmap of fold-differences between grouped samples of PG and PGF. Lower right: MA plot showing log2 fold change against average normalised expression (significant differential expression coloured red). B: Transcriptomic analysis of selected genes with added formate; ETC – electron transport chain; Secondary metabolism; genes involved in production of specific metabolites such as inositol.

Figure S5

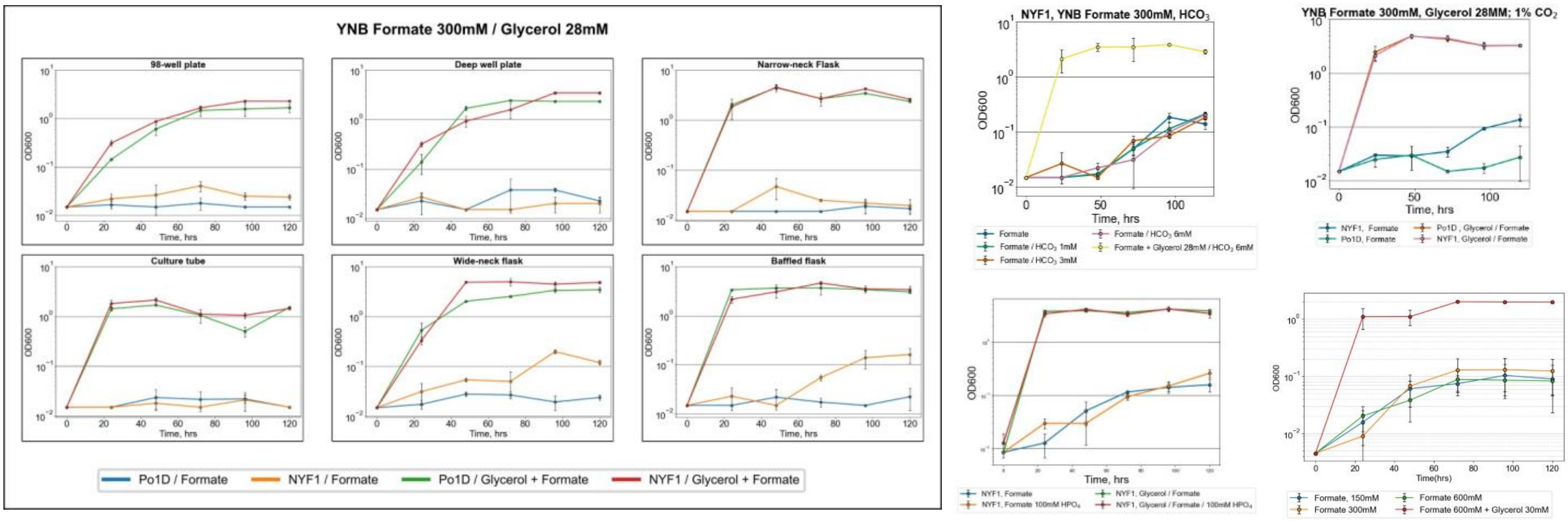

Figure S5: Left - summarised culture method experiments in 300rpm shaking incubator (n=2, biological duplicates). Upper right – high CO2 experiments with bicarbonate (left) and CO2 (right). Lower right: Growth with and without added phosphate (left) and at different formate concentrations (right). All data are biological duplicates and presented as means +/1 SD.

Figure S6

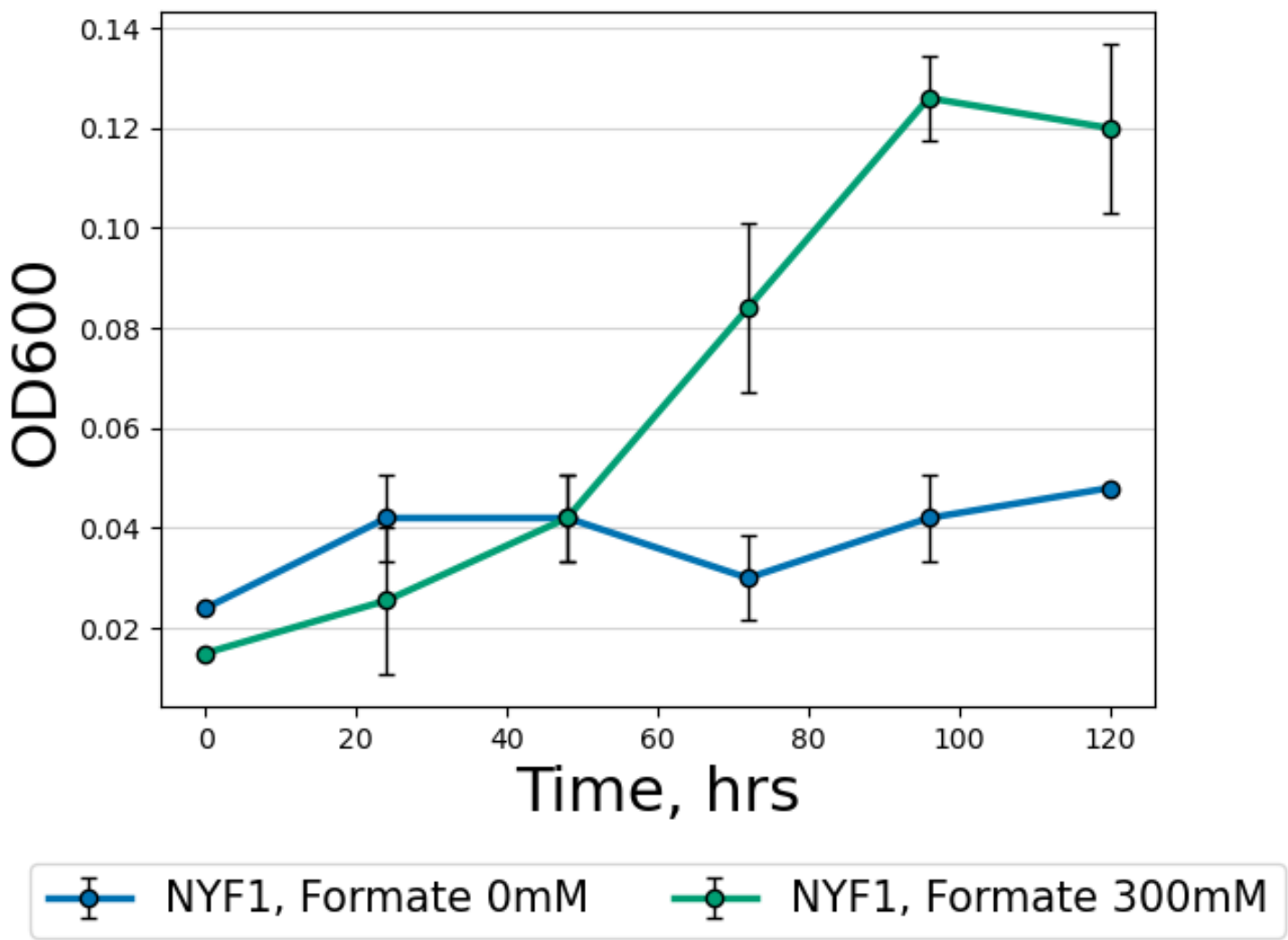

Supplemental figure S6: growth of NYF1 strain in media with and without carbon source, presented as mean +/- SD (formate 300mM, n=2, biological duplicates)

Figure S7

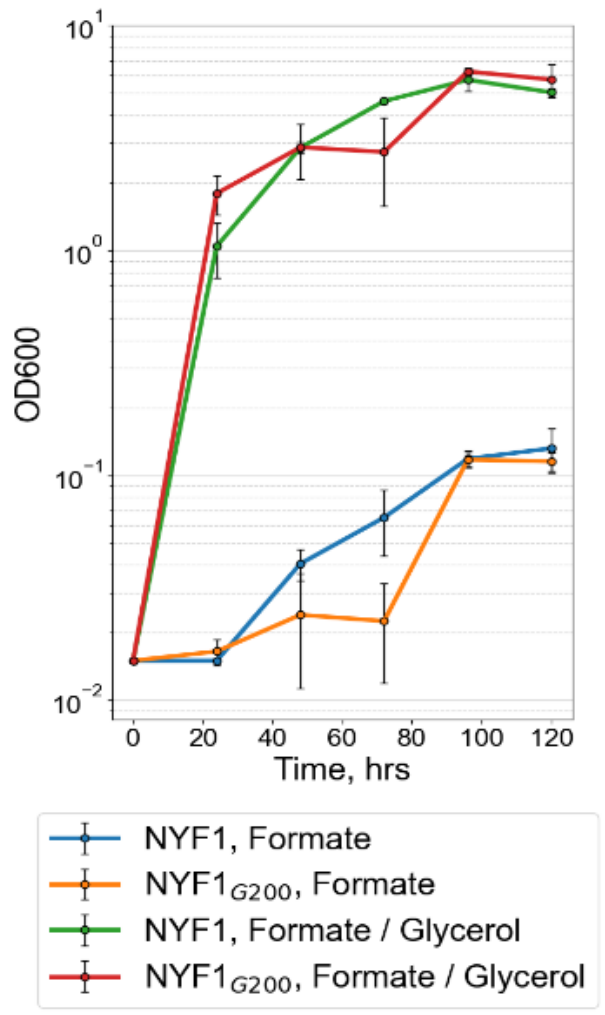

Supplemental figure S7: Strain NYF1 maintains formatotrophy after repeated culture in non-selective media (n=2, biological duplicates, presented as mean +/- SD).

Figure S8

Effect	Codon change	Gene ID	Annotation summary
Frame_shift	gtt/	YALI0_F15015g	--
Frame_shift	aac/	YALI0_C23617g	Glyoxylate pathway regulator (GPR1)
Non_synonymous_coding	cAt/cTt	YALI0_B04598g	Autophagy-related protein 11 (ATG11)
Non_synonymous_coding	Gct/Act	YALI0_B18810g	Clustered mitochondria protein homolog (CLU1)
Stop_gained	Cag/Tag	YALI0_B22176g	C2H2 finger domain transcription factor (USV1)

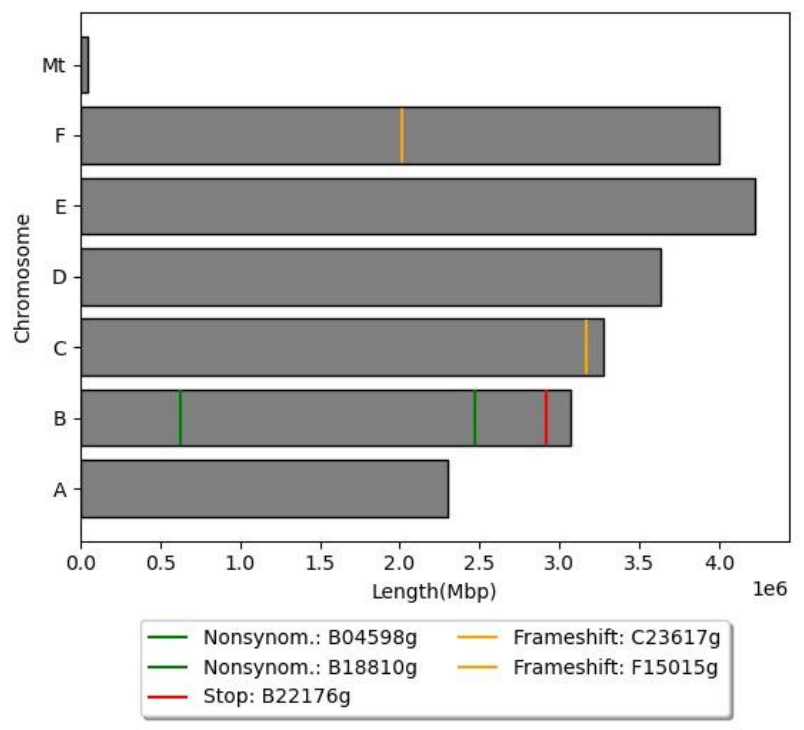

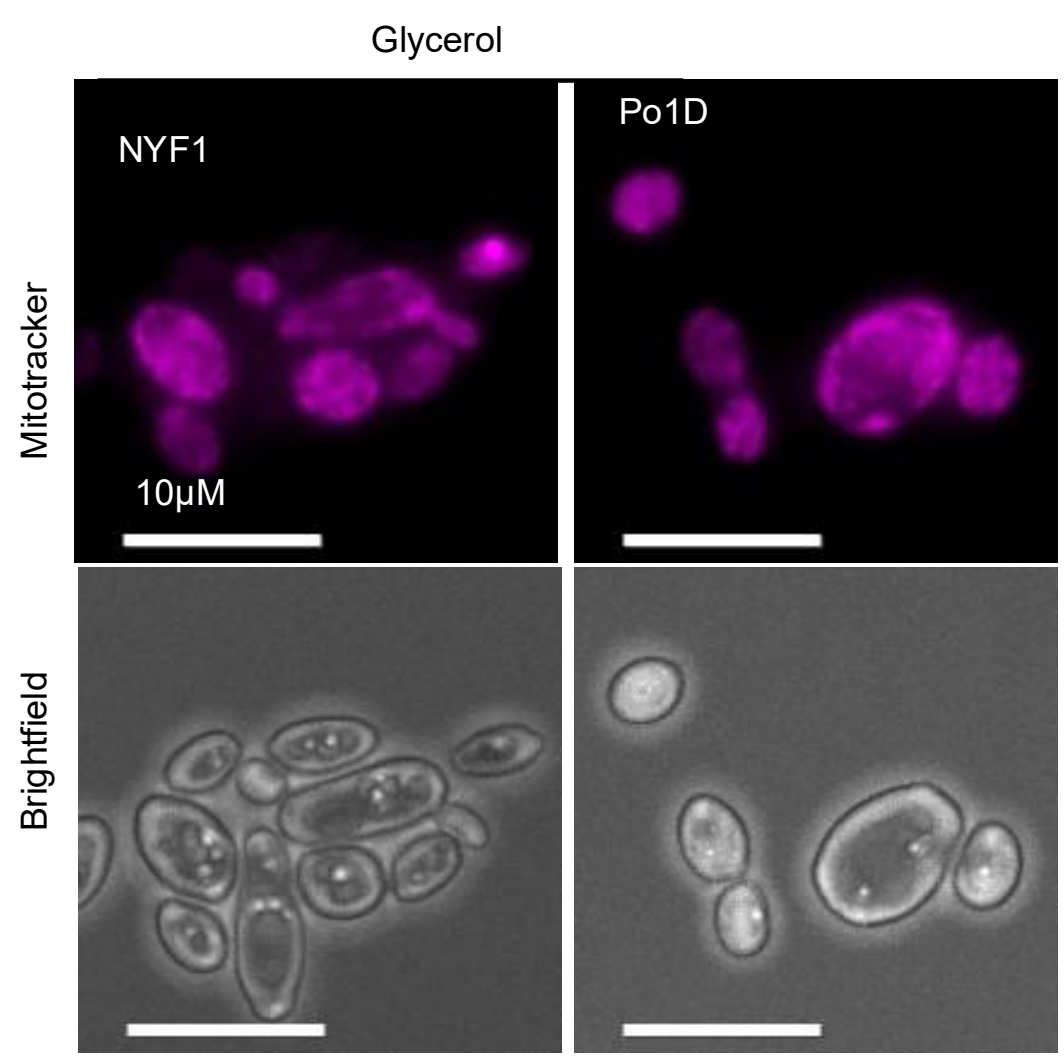

Supplemental figure S8: CDS mutations details and loci (upper and lower). Mitotracker stained cells (Right)

Figure S9

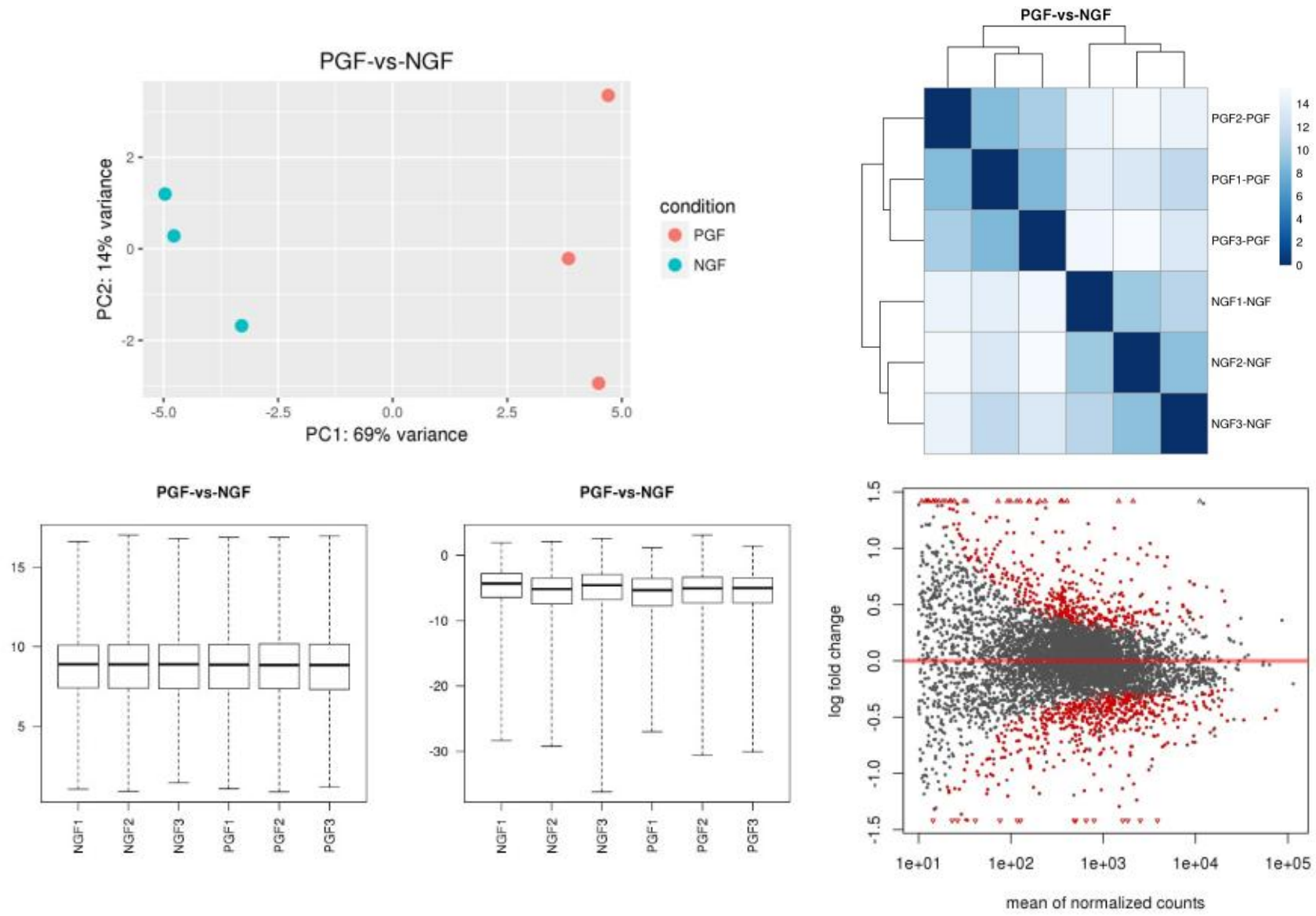

Supplemental figure S9: PCA of Po1D grown in glycerol and formate vs NYF1 grown in glycerol and formate (upper left). Lower left: box plot of normalised (left) and raw (right) average gene expression values. Upper right: heatmap of fold-differences between grouped samples of PGF and NGF. Lower right: MA plot showing log2 fold change against average normalised expression (significant differential expression coloured red)

Figure S10

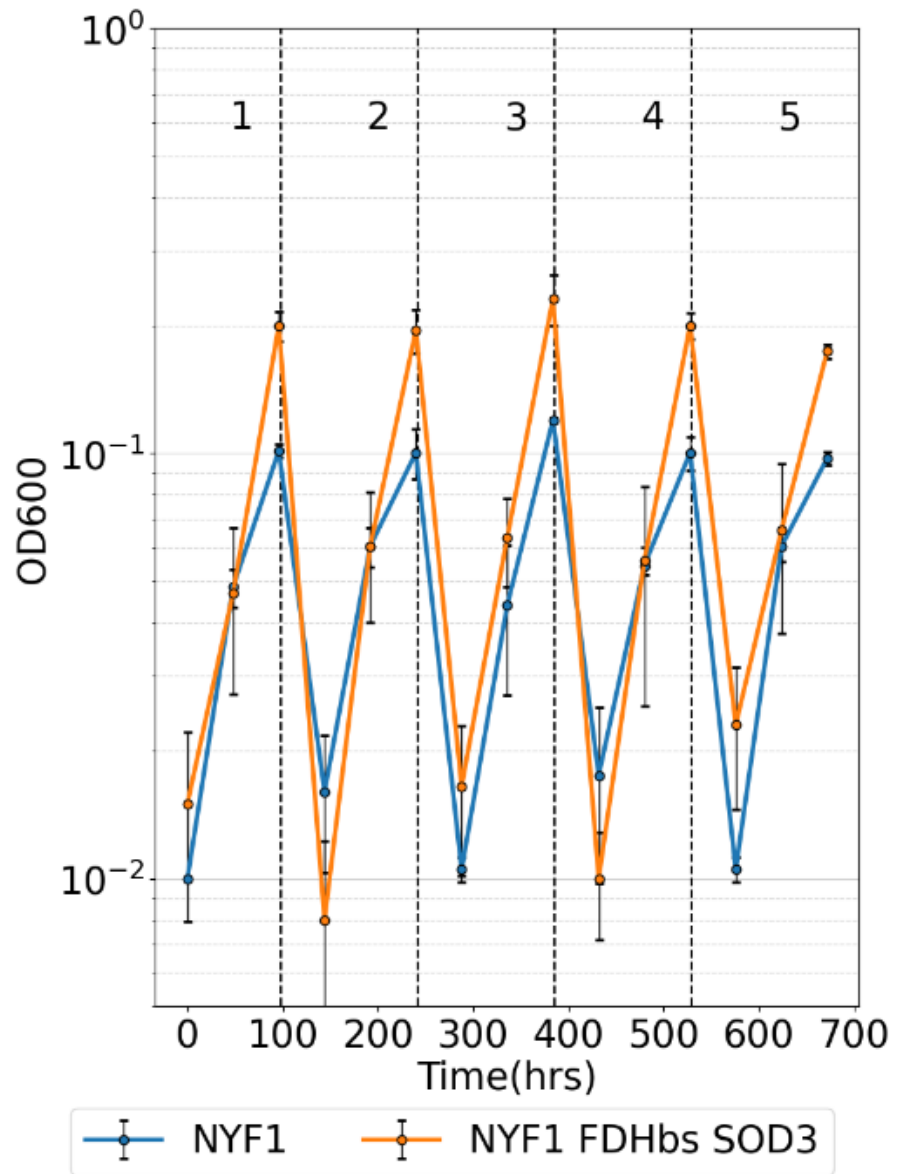

Supplemental figure S10: NYF1 grown over multiple passages in formate. Numbers indicate passages – cells were regrown at 120 hours, corresponding to T=0 of the next passage. (N=2, biological duplicates; data presented as mean +/- SD).

Figure S11

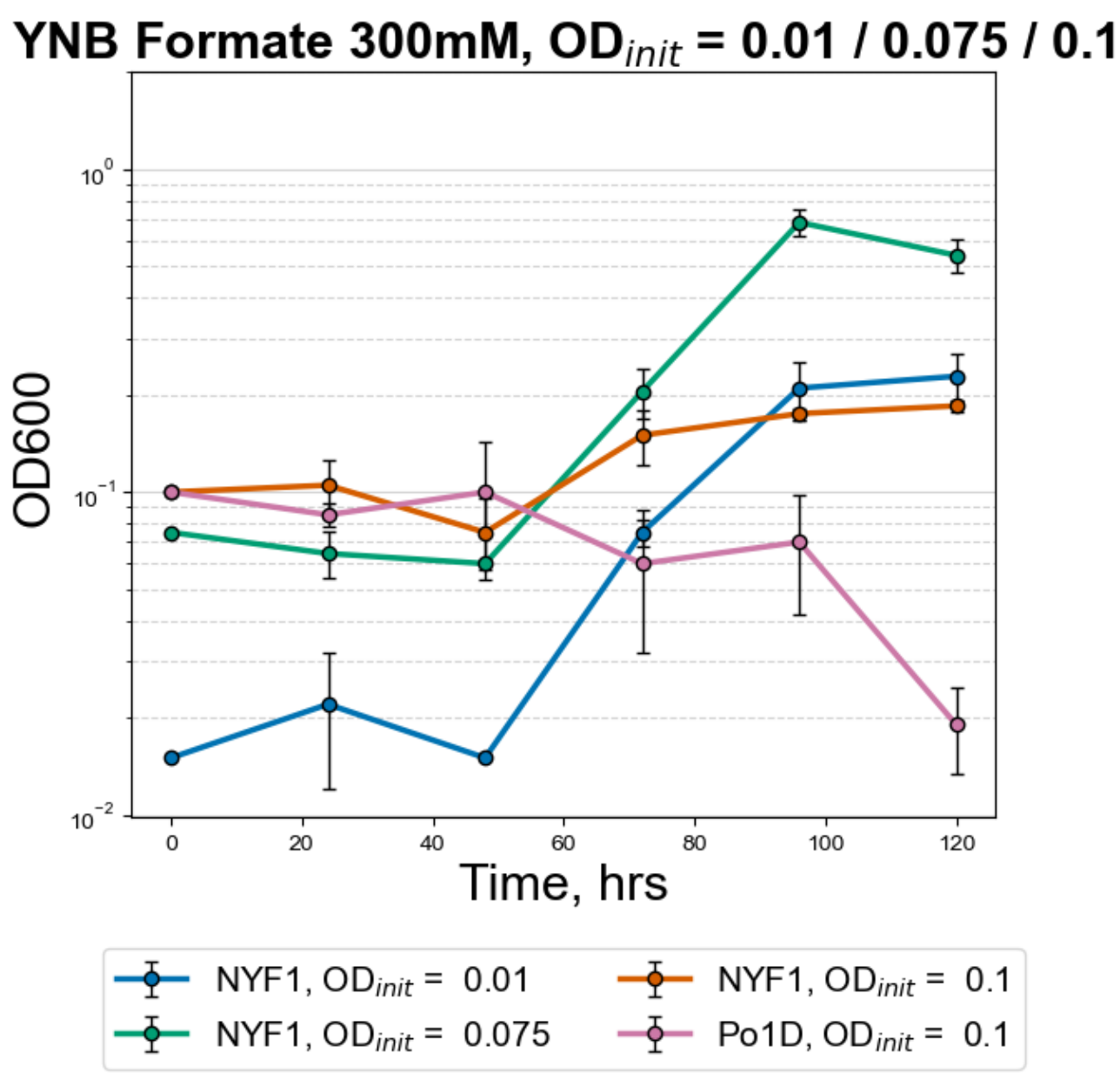

Supplemental figure S11: growth of NYF1 at differing initial ODs. . N=2 (biological duplicates, data presented as mean +/1 SD).

Figure S12

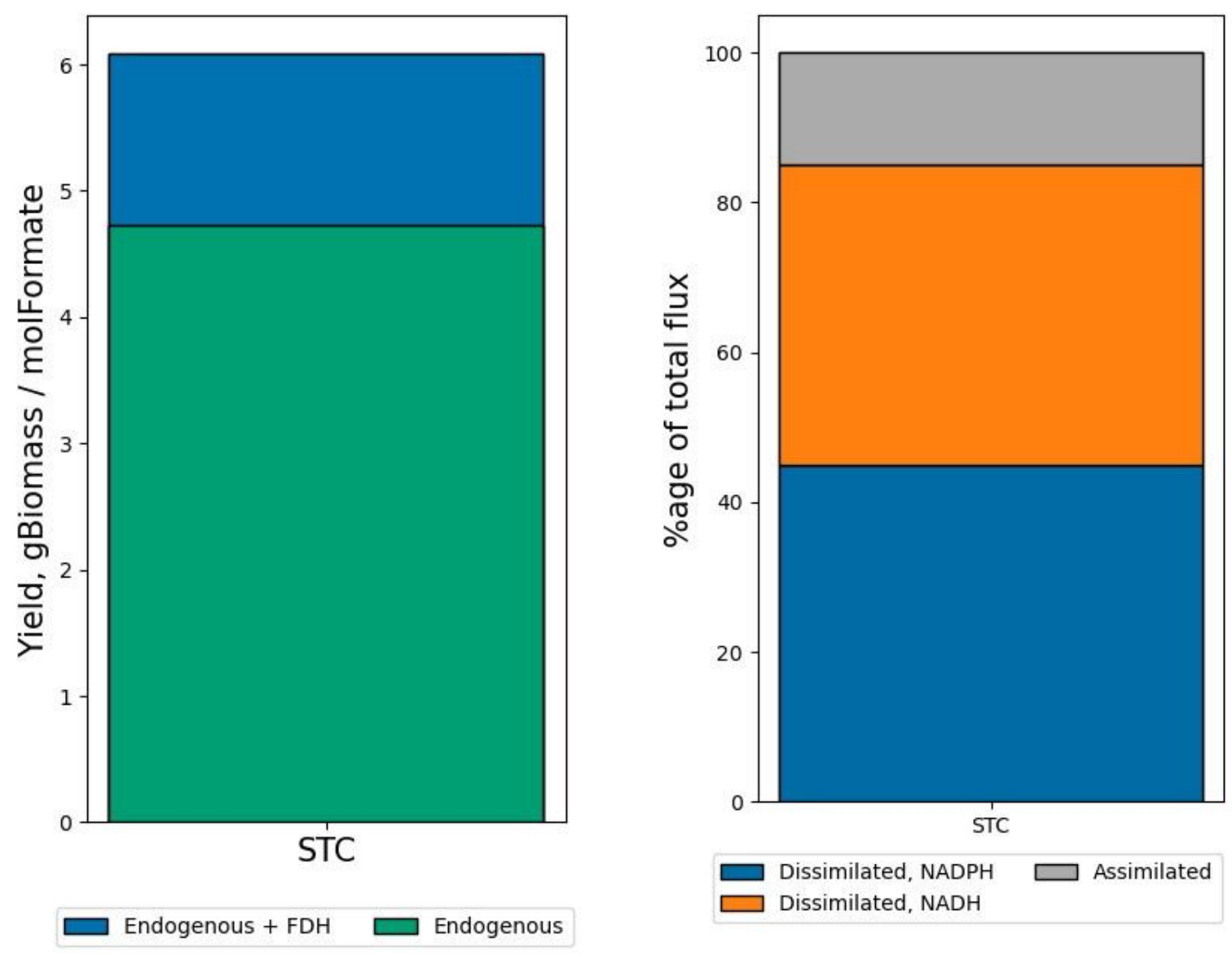

Supplemental figure S12: (left) Simulated biomass yields on formate via the Serine-Threonine cycle with (blue) and without (teal) expression of FDHbs. Right: distribution of flux between assimilation or dissimilation to NADH / NADPH during growth on the ST-cycle

Figure S13

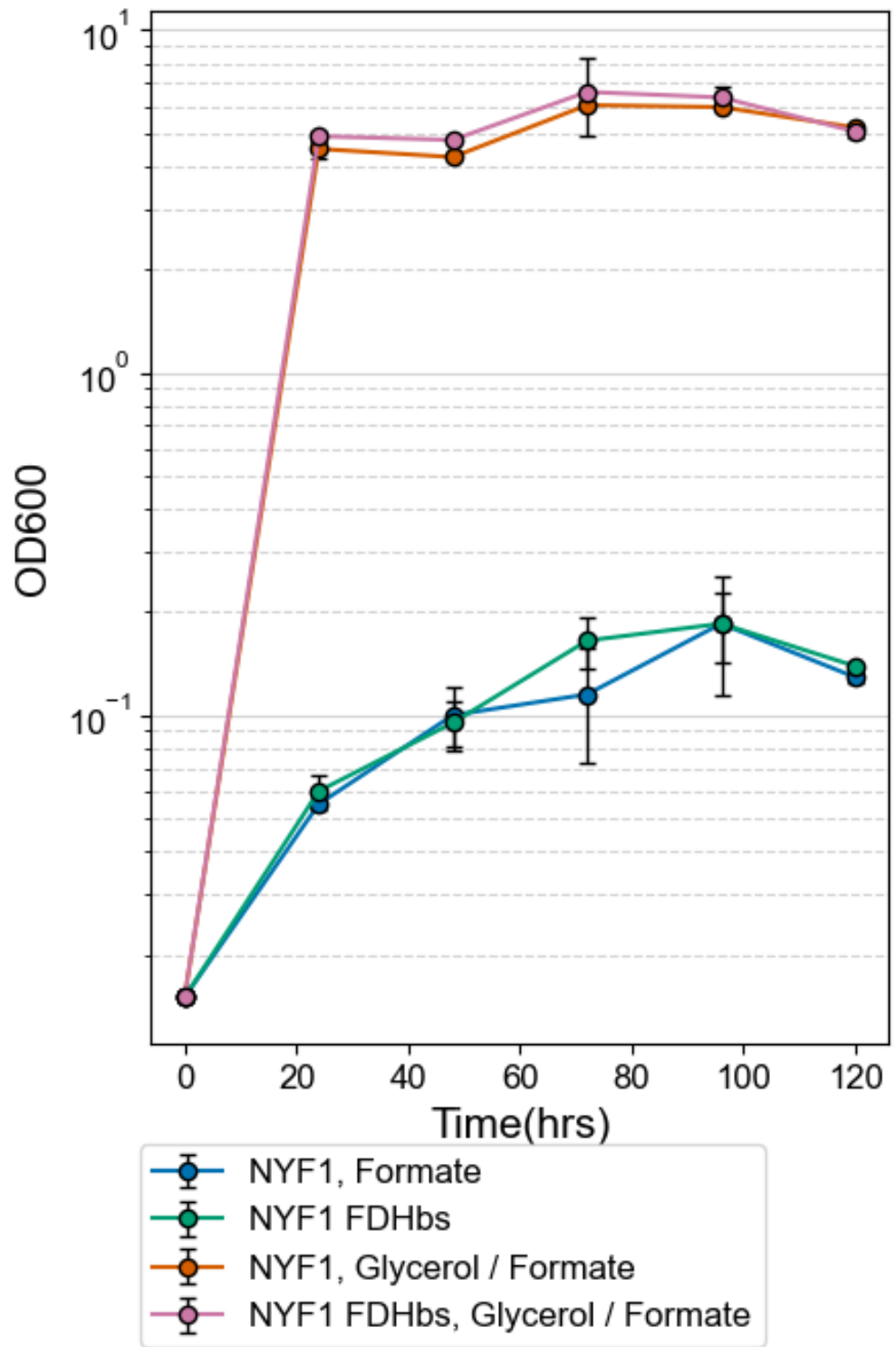

Supplemental figure S13: Growth curve of NYF1 (blue, orange) and with FDH from *B. stabilis* (teal, pink). N=2 (biological duplicates, data presented as mean +/- SD).

Figure S14

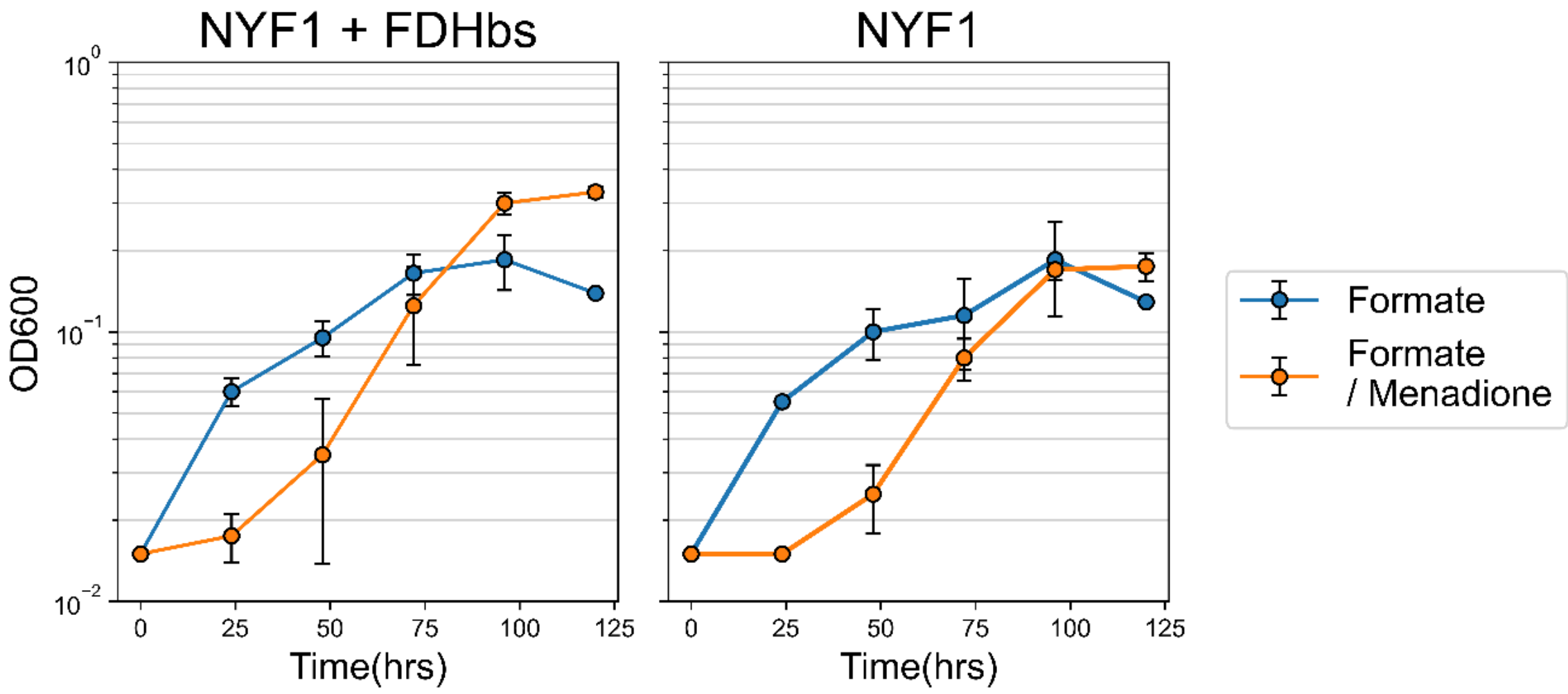

Supplemental figure S14: NYF1 precultured with menadione (teal) reaches a higher final OD than NYF1 precultured in ordinary media (blue) when FDH is expressed. N=2 (biological duplicates, data presented as mean +/- SD).

Figure S15

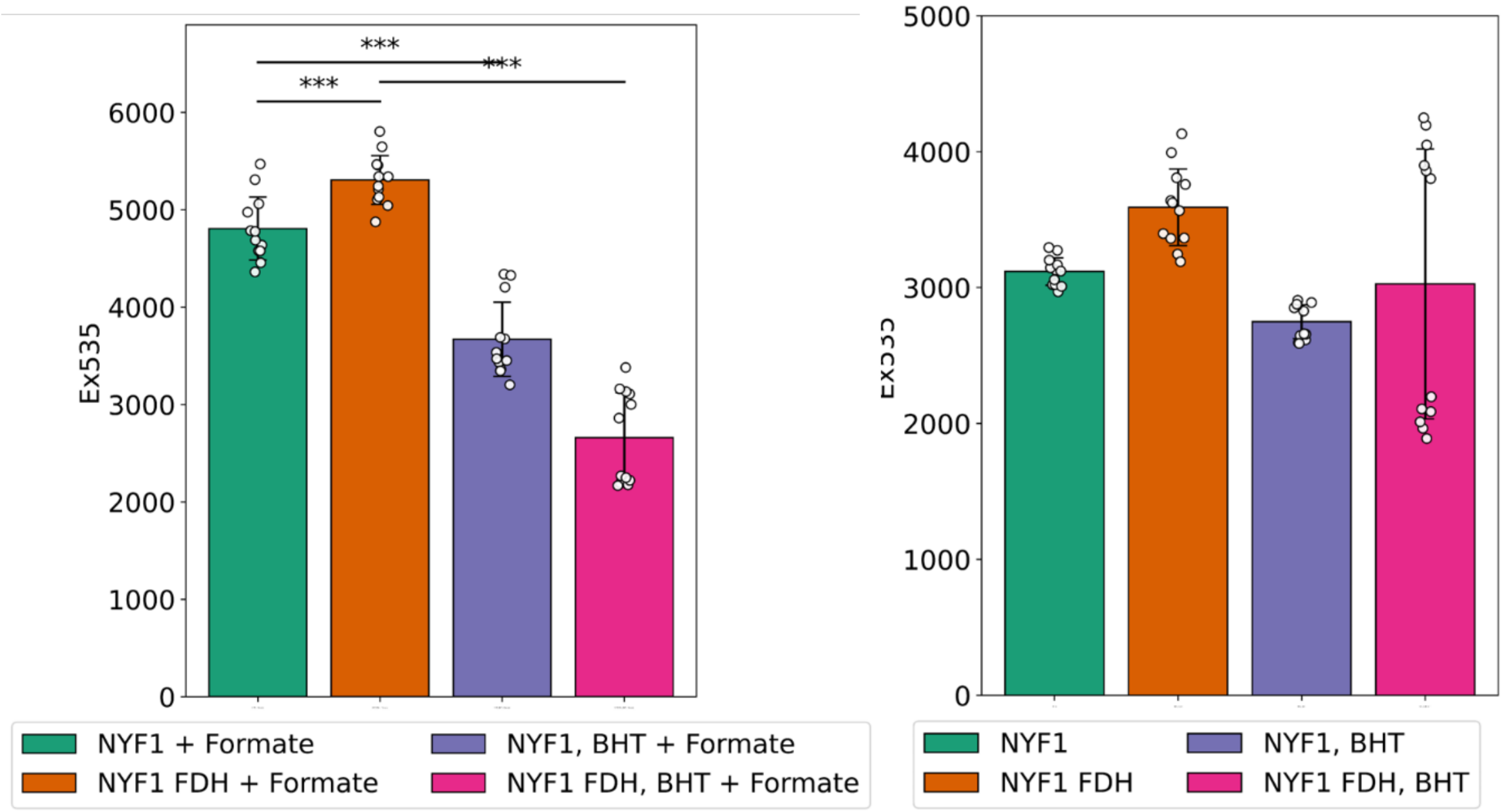

Supplementary figure S15: Left: DCFDA assay of strains NYF1 and NYF1 + FDHbs growing on glycerol + formate, with and without added BHT. Right: positive controls showing ROS production in NYF1 and FDH during growth on glycerol / glycerol + BHT. Data are presented as means with +/- SD. Statistical significance of p-values from 2-tailed T-tests summarised as * = $p>0.05$, ** = $p>0.01$, ***= $p>0.005$.

Figure S16

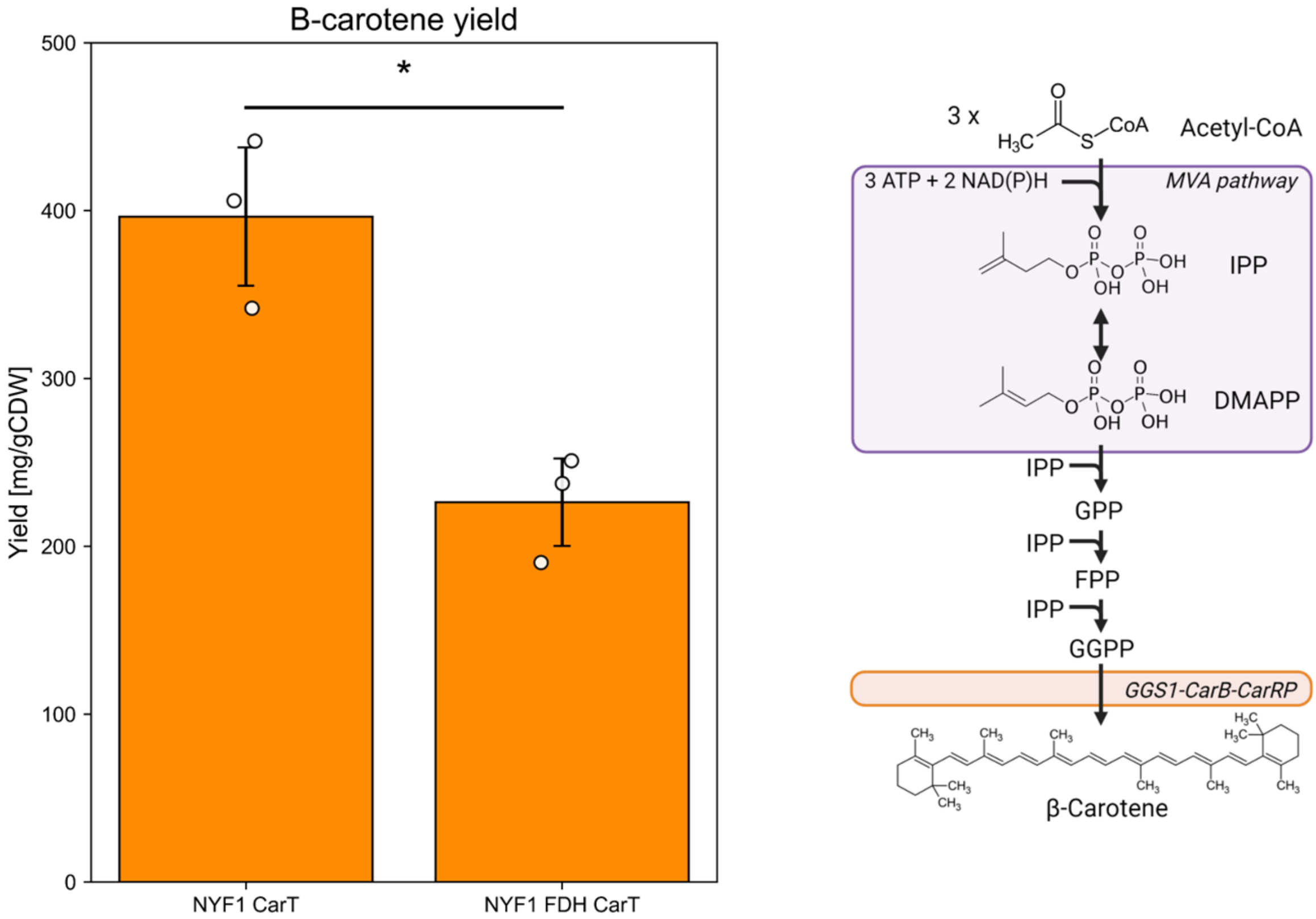

Supplementary figure S16: Left - Carotene accumulation (gCarotene/gCDW). N=3 (biological triplciates, data presented as mean +/- SD) with significance in 2-sample 2-tailed t-tests summarised for clarity as * = p>0.05, ** = p>0.01, ***=p>0.005. Right - pathway schematic showing carotenogenesis. Coloured boxes indicate summarised pathway and italics genes involve

Supplementary Note 1

The reductive glycine pathway synthesises glycine from formate and CO2, which would give rise to single labelled glycine and twice labelled serine. However, the serine-threonine cycle should not synthesise large amounts of glycine from formate, instead creating once-labelled serine.   Neither serine nor pyruvate showed substantial m+2 labelling – likewise, GPE (formed by decarboxylation of serine and ligation to glycerol-phosphate) was only labelled once. GPI, formed by ligation of inositol to glycerol phosphate, was not labelled. Aspartate and asparagine both show m+1 labelling patterns – consistent with carboxylation of pyruvate derived from once-labelled serine. Threonine and isoleucine are also only labelled once (fig. 2C). Cleavage of once-labelled threonine should then regenerate unlabelled glycine and produce once-labelled acetyl-CoA. Indeed, small quantities of citrate are labelled once, as well as alpha-ketoglutarate derived amino acids (glutamate and glutamine), which could stem from once-labelled acetyl-CoA entering the TCA cycle. Palmitate is synthesised from acetyl-CoA monomers and is also once labelled. Surprisingly, no m+2 or greater palmitate was detected –*Y. lipolytica* tends to accumulate large amounts of acetyl-CoA on glycerol and this could therefore ‘dilute’ the labelled pool palmitate precursors. Furthermore, glutathione

(GSH) is largely labelled at m+1 and m+2. Glutathione is synthesised via glutamate ligation to cysteine followed by addition of glycine. The lack of large quantities of m+3 labelled GSH – given serine and c5 labelling patterns - further suggests that glycine is not primarily synthesised from formate (Fig. 2C).

These results also show that large amounts of methionine and histidine were once-labelled. Biosynthesis of these both run via a step including 10-formyl THF, which is derived from 5,10 methylene-THF (Fig 1. C). Furthermore, both AMP and xanthine showed large proportions of m+1 and m+2 labels. These are synthesised via purine biosynthesis, involving incorporation of glycine and 2 methylation steps using 10-fTHF. Double labelling of these two metabolites, as well as m+1 labelling of His and Met, shows that formate ligation to THF occurs during formate assimilation. Moreover, the lack of much m+3 labelling in both further shows the lack of glycine synthesis from formate. Labelling was not observed in glucose or inositol, or the PPP-derived amino acid phenylalanine (supplementary figure 3).

Supplementary Note 2

mRNA sequencing also showed several changes in glycolytic and electron transport gene expression. In general, glycolytic gene expression is correlated with growth rate [1], which may explain why there seemed to be a global decrease in glycolysis expression (Fig. 2B). However, the serine biosynthetic gene phosphohomoserine dehydrogenase (Ser 33) was also downregulated (fig. 2C). Ser33 is regulated in response to serine demand in *S. cerevisiae* and so lower expression would suggest greater serine biosynthesis from formate attachment to glycine, as opposed to from glycolytic intermediates. Ser33 has also been proposed to act as an alpha-ketoglutarate reductase – given that Krebs cycle expression clearly falls in *Y. lipolytica*, it is possible that Ser33 expression responds to falling TCA flux[2] (fig. 2B).

Electron transport genes are downregulated upon heat stress in *S. cerevisiae* as cells attempt to minimise ROS production by electrons leaking from the transport chain[3]. We also observe downregulation of ETC genes such as cytochrome oxidases (COX13, 12, 6 and 5) cytochromes (CYC1) and the transmembrane H+/ATP exchanger PMA1. It is therefore tempting to speculate that *Y. lipolytica* reduces electron transport chain expression to cope with higher oxidative stress emerging complex 4 inhibition by formate. However, we do not observe upregulation of the alternative oxidase AOX1, which is commonly used to reduce electron leakage during ETC inhibition by cyanide, another complex IV inhibitor [4].

Finally, we also observed a general increase in NADH-dependent dehydrogenase expression. Notably, multiple ADH12 and ALD5 homologues were upregulated. These are the main enzymes responsible for converting acetaldehyde to acetate during growth on ethanol in *S. cerevisiae*, and so may help drive acetaldehyde conversion produced by threonine cleavage in the ST-cycle[5]. However, aldehyde dehydrogenases are also involved in oxidative stress resistance mechanisms and so may be upregulated in response to the observed increase in ROS production[6].

1. Coordination of Growth Rate, Cell Cycle, Stress Response, and Metabolic Activity in Yeast | Molecular Biology of the Cell. https://www.molbiolcell.org/doi/full/10.1091/mbc.e07-08-0779.

2. Albers, E., Laizé, V., Blomberg, A., Hohmann, S. & Gustafsson, L. Ser3p (Yer081wp) and Ser33p (Yil074cp) Are Phosphoglycerate Dehydrogenases in Saccharomyces cerevisiae. *Journal of Biological Chemistry* **278**, 10264–10272 (2003).

3. Sakaki, K., Tashiro, K., Kuhara, S. & Mihara, K. Response of Genes Associated with Mitochondrial Function to Mild Heat Stress in Yeast Saccharomyces cerevisiae. *The Journal of Biochemistry* **134**, 373–384 (2003).

4. Veiga, A., Arrabaca, J. & Loureirodias, M. Cyanide-resistant respiration, a very frequent metabolic pathway in yeasts. *FEMS Yeast Research* **3**, 239–245 (2003).

5. Aranda, A. & del Olmo Ml, M. lí. Response to acetaldehyde stress in the yeast Saccharomyces cerevisiae involves a strain-dependent regulation of several ALD genes and is mediated by the general stress response pathway. *Yeast* **20**, 747–759 (2003).

6. Singh, S. *et al.* Aldehyde dehydrogenases in cellular responses to oxidative/electrophilicstress. *Free Radical Biology and Medicine* **56**, 89–101 (2013).

Supplementary Note 3

In order to rule out an epigenetic basis for formatotrophy, we grew NYF1 for roughly 200 generations in nonselective media (YPD +2% glucose). Epigenetic silencing of uracil biosynthesis in *S. cerevisiae* is reversed after roughly 90 generations[1], and so we reasoned this process could 're-set' NYF1 to a non-formatotrophic phenotype by reversing supporting epigenetic changes. NYF1 maintained growth on formate after 200 generations, suggesting that the novel phenotype was genomically encoded.

1. Rando, O. J. & Verstrepen, K. J. Timescales of Genetic and Epigenetic Inheritance. *Cell* **128**, 655–668 (2007).

Supplementary Note 4

FDHbs expression increased ROS production even in strain NYF1 grown in glycerol. This could be the result of naturally-produced formate being converted to CO2 and NADPH by FDHbs. Physiological formate production is possible via the normal operation of the C1 cycle, where mitochondrially - produced serine is converted into glycine and 5,10 mTHF. 5,10mTHF is then converted back to formate by C1THFS1[1]. Even small amounts of formate are known to stimulate ROS production[2], and so formate conversion to NADPH by FDHbs may be sufficient to stimulate reductive stress-mediate ROS production.

1. Piper, M. D., Hong, S.-P., Ball, G. E. & Dawes, I. W. Regulation of the Balance of One-carbon Metabolism in*Saccharomyces cerevisiae* *. *Journal of Biological Chemistry* **275**, 30987–30995 (2000).

2. Young, A., Gardiner, D., Brosnan, M. E., Brosnan, J. T. & Mailloux, R. J. Physiological levels of formate activate mitochondrial superoxide/hydrogen peroxide release from mouse liver mitochondria. *FEBS Lett* **591**, 2426–2438 (2017).

Supplementary Data Table 1: Strains used in this study

Strain Name	Phenotype
Po1D	Po1D Ura / Leu deletion mutant with auxotorphies reversed by transformation with JMP URA / LEU plasmids
NYF1	Po1D evolved to grow on formate (this study)
NYF1 FDHbs	NYF1 plasmid expressing B. stabilis FDH under constitutive Tef promoter and NAT resistance (JMP series of vectors)
NYF1 SOD3	NYF1 transformed with heterologous SOD3 (SuperOxide Dismutase 3) under Tef promoter and Hyg resistance(Yalicraft / YaTK series of vectors)
NYF1 SOD3 FDH	NYF1 FDH transformed with heterologous SOD3 (SuperOxide Dismutase 3)under Tef promoter and Hyg resistance(Yalicraft / YaTK series of vectors)
NYF1 SOD2	NYF1 transformed with heterologous SOD2 (SuperOxide Dismutase 2) under Tef promoter and Hyg resistance(Yalicraft / YaTK series of vectors)
NYF1 SOD2 FDH	NYF1 FDH transformed with heterologous SOD2 (SuperOxide Dismutase 2) under Tef promoter and Hyg resistance(Yalicraft / YaTK series of vectors)
NYF1 GSR	NYF1 transformed with heterologous GSR1 (Glutathione Reductase 1) under Tef promoter and Hyg resistance(Yalicraft / YaTK series of vectors)
NYF1 GSR FDH	NYF1 FDH transformed with heterologous GSR1 (Glutathione Reductase 1) under Tef promoter and Hyg resistance(Yalicraft / YaTK series of vectors)
NYF1 CarT	NYF1 transformed with heterologous GGS1, CarB and CarRP under Tef8UAS promotersr and Hyg resistance(JMP series of vectors)
NYF1 FDHbs CarT	NYF1 FDH transformed with heterologous GGS1, CarB and CarRP under Tef8UAS promotersr and Hyg resistance(JMP series of vectors)

Supplementary Data Table 2: Gene sequences of overexpressed genes

Gene name	Sequence
SOD2	ATGCTCGCCCGAAACACTCTTCGACAGACCATTCGAAAGGCCCAGCC<br>CATGGCCGGCCGAACCCTGACCACCGCCGTTCTGCCCAAGCTGGACT<br>ACGACTTTGGCGCCCTGGAGCCCTTTGTGGCCGGCCAGATCAACGAG<br>ATCCACTACACCAAGCACCACCAGACCTACGTCAACTCGTTCAACGC<br>CGCCTCCGAGCAGCTGGCCTCCGCCGAGAAGGACGGAGACCTCAAG<br>AAGATTGTCGAGCTGCAGAACCTGATCAACTTCCACGGCGGAGGACA

	CATCAACCACTCTCTGTGGTGGAAGAACCTGGCTCCCAAGGGCCAGG<br>GTGGAGGCGAGCTGCCTTCTCCCGACTCGCCTCTCGGCAAGGCCATC<br>AAGGAGCAGTGGGGAGACGCCAACAAGCTGATCGAAACCACCAACG<br>CTGCTCTCGCCGGTGTCCAGGGCTCCGGATGGGCCTGGATCGTCAAG<br>AACAAGCTCACCGGTGACATCGAGGTCATCACCAAGCAGAACCAGG<br>ACCCCGTGGCCGGCAACCACATCCCTCTCGTCGGTATCGACGCCTGG<br>GAGCACGCCTACTACCTCCAGTACAAGAACGTCAAGGCTGACTACTT<br>CAAGGCCATCTGGAACGTCATCAACTGGCAGGAGGCTGAGAAGCGA<br>TTCCTCGGTGAGGCCAAGTTTGTCAACGGCCAGTAA
SOD3	ATGCATGGTCTCAATCAGTCAGCATCAATGCTCCGGTCTAAGTGGATG<br>GAGGTCGGTACAGTACCTGGAATAGTCCCTTGTCTCTGTGTGACCAG<br>ACATGACCAAATATGGTACAAATCTATTGGAAGAGCAACTTGATTGAC<br>TCACCCGACTCAATTGATTCACGTGGTGCCTAGTCCGTCTGTTAACCG<br>GGTCTCTGTCTAAACTAATCGAGTCCCTAAGTGATTCCCGGGACTCGA<br>CGTGTCTAGCCCTGTCAACTATCGGGTCTGCTCTCCCTGGTTTAGATC<br>CCGTCAATCACTCTGCCTCTGCTCTCGTACTAACCTAGGCCCTCGAGC<br>CCCAGATCTCCGCCCAGATCATGGAGATCCATCACTCCAAGCACCACC<br>AGACCTACGTGAACAACCTCAATGCTGCCACCAAGCAGCTGCAGGA<br>GGCTGTCGCCGCCAACGACGTGGTCAGACAAATTGCTCTGCAGAAG<br>GCCATCAACTTCAACGGAGGAGGCCATGTCAACCACTCCATTTTCTG<br>GAAGTCACTGACCCCCTCGTCTTCCGACGGAGCCAAGGGAGGCGCG<br>AAACTCAAGGAGGAGATTGCCAAGCAGTTTGGCTCTCTGGATGAGTT<br>CAAAGCCAAGCTCAAGGCTGATCTTCTGGCTCTCCAGGGCTCTGGTT<br>GGGCTTGGCTCGTGTCTTACCCCGACGGAACTCTCAAGATCGAGGTC<br>ACCAGCAACCAGGACGCTATTTCGGCCCCCGGAGCCGTCACTCTGCT<br>GGGAATCGACTTTTGGGAGCACGCCTACTACATCCAGTACTACAACA<br>ACAAGGCCGCCTACTTTGACAACCTCTGGGAGGTGCTCAACTGGGAC<br>TTTGCCGAGAAACAGTGGGCCCAGCGACCTCTGGCCAAGTACTAA
GSR1	ATGCATGGTCTCAATCAGTCAGCATCAATGCTCCGGTCTAAGTGGATG<br>GAGGTCGGTACAGTACCTGGAATAGTCCCTTGTCTCTGTGTGACCAG<br>ACATGACCAAATATGGTACAAATCTATTGGAAGAGCAACTTGATTGAC<br>TCACCCGACTCAATTGATTCACGTGGTGCCTAGTCCGTCTGTTAACCG<br>GGTCTCTGTCTAAACTAATCGAGTCCCTAAGTGATTCCCGGGACTCGA<br>CGTGTCTAGCCCTGTCAACTATCGGGTCTGCTCTCCCTGGTTTAGATC<br>CCGTCAATCACTCTGCCTCTGCTCTCGTACTAACCTAGGCCCTCGAGC<br>CCCAGATCTCCGCCCAGATCATGGAGATCCATCACTCCAAGCACCACC<br>AGACCTACGTGAACAACCTCAATGCTGCCACCAAGCAGCTGCAGGA<br>GGCTGTCGCCGCCAACGACGTGGTCAGACAAATTGCTCTGCAGAAG<br>GCCATCAACTTCAACGGAGGAGGCCATGTCAACCACTCCATTTTCTG<br>GAAGTCACTGACCCCCTCGTCTTCCGACGGAGCCAAGGGAGGCGCG<br>AAACTCAAGGAGGAGATTGCCAAGCAGTTTGGCTCTCTGGATGAGTT<br>CAAAGCCAAGCTCAAGGCTGATCTTCTGGCTCTCCAGGGCTCTGGTT<br>GGGCTTGGCTCGTGTCTTACCCCGACGGAACTCTCAAGATCGAGGTC<br>ACCAGCAACCAGGACGCTATTTCGGCCCCCGGAGCCGTCACTCTGCT<br>GGGAATCGACTTTTGGGAGCACGCCTACTACATCCAGTACTACAACA<br>ACAAGGCCGCCTACTTTGACAACCTCTGGGAGGTGCTCAACTGGGAC<br>TTTGCCGAGAAACAGTGGGCCCAGCGACCTCTGGCCAAGTACTAA
FDHbs	ATGGCCACCGTGCTCTGTGTCCTGTACCCCGACCCTGTGGATGGATAC<br>CCTCCCCACTACGTTCGAGACACCATCCCTGTGATTACTCGATACGCC<br>GATGGTCAGACCGCTCCTACTCCCGCCGGCCCTCCCGGCTTCCGACC

	CGGCGAGCTGGTTGGCTCTGTGTCCGGTGCCCTGGGCCTCCGAGGAT<br>ACCTGGAGGCTCACGGACATACCCTCATTGTGACTTCTGACAAGGAT<br>GGTCCTGACTCCGAGTTCGAGCGACGACTGCCTGACGCTGATGTGGT<br>CATCTCCCAGCCTTTTTGGCCCGCCTACCTCACCGCTGAGCGAATTGC<br>CCGAGCTCCTAAGCTGCGACTCGCTCTGACTGCTGGCATCGGATCGG<br>ACCACGTCGACCTGGATGCCGCTGCCCGAGCCCATATCACCGTCGCT<br>GAGGTTACTGGATCGAACTCTATTTCCGTCGCCGAGCACGTTGTGATG<br>ACCACTCTCGCTCTGGTTCGAAACTACCTGCCTTCTCACGCCATCGCT<br>CAGCAGGGCGGATGGAACATTGCCGACTGTGTCTCGCGATCTTACGA<br>TGTTGAGGGAATGCACTTCGGTACCGTGGGTGCTGGTCGAATTGGTC<br>TCGCTGTCCTGCGACGACTCAAGCCTTTTGGACTCCACCTGCATTACA<br>CCCAGCGACACCGACTGGACGCTGCCATCGAGCAGGAGCTCGGACT<br>GACTTACCATGCCGACCCCGCTTCGCTCGCTGCCGCTGTGGATATCGT<br>CAACCTGCAGATTCCTCTCTACCCCTCTACCGAGCACCTGTTCGACGC<br>CGCTATGATCGCCCGAATGAAGCGAGGCGCTTACCTGATTAACACCGC<br>CCGAGCTAAGCTCGTTGACCGAGATGCCGTCGTTCGAGCTGTGACTT<br>CTGGCCATCTGGCCGGATACGGTGGCGACGTGTGGTTCCCTCAGCCT<br>GCTCCCGCTGATCACCCTTGGCGAGCCATGCCCTTTAACGGTATGACC<br>CCTCATATTTCCGGCACTTCCCTGTCGGCTCAGGCTCGATACGCCGCT<br>GGTACCCTGGAGATCCTCCAGTGCTGGTTTGACGGCCGACCCATCCG<br>AAACGAGTACCTGATTGTCGACGGAGGTACCCTCGCCGGAACTGGTG<br>CTCAGTCTTACCGACTGACCTAGTAA
GGS1	ATGGATTATAACAGCGCGGATTTCAAGGAGATATGGGGCAAGGCCGC<br>CGACACCGCGCTGCTGGGACCGTACAACTACCTCGCCAACAACCGG<br>GGCCACAACATCAGAGAACACTTGATCGCAGCGTTCGGAGCGGTTAT<br>CAAGGTGGACAAGAGCGATCTCGAGACTATTTCGCACATCACCAAGA<br>TTTTGCATAACTCGTCGCTGCTTGTTGATGACGTGGAAGACAACTCGA<br>TGCTCCGACGAGGCCTGCCGGCAGCCCATTGTCTGTTTGGAGTCCCC<br>CAAACCATCAACTCCGCCAACTACATGTACTTTGTGGCTCTGCAGGA<br>GGTGCTCAAGCTCAAGTCTTATGATGCCGTGTCCATTTTCACCGAGGA<br>AATGATCAACTTGCATAGAGGTCAGGGTATGGATCTCTACTGGAGAGA<br>AACACTCACTTGCCCCTCGGAAGACGAGTATCTGGAGATGGTGGTGC<br>ACAAGACCGGTGGACTGTTTCGGCTGGCTCTGAGACTTATGCTGTCG<br>GTGGCATCGAAACAGGAGGACCATGAAAAGATCAACTTTGATCTCAC<br>ACACCTTACCGACACACTGGGAGTCATTTACCAGATTCTGGATGATTA<br>CCTCAACCTGCAGTCCACGGAATTGACCGAGAACAAGGGATTCTGCG<br>AAGATATCAGCGAAGGAAAGTTTTCGTTTCCGCTGATTCACAGCATAC<br>GCACCAACCCGGATAACCACGAGATTCTCAACATTCTCAAACAGCGA<br>ACAAGCGACGCTTCACTCAAAAAGTACGCCGTGGACTACATGAGAAC<br>AGAAACCAAGAGTTTCGACTACTGCCTCAAGAGGATACAGGCCATGT<br>CACTCAAGGCAAGTTCGTACATTGATGATCTAGCAGCAGCTGGCCAC<br>GATGTCTCCAAGCTACGAGCCATTTTGCATTATTTTGTGTCCACCTCTG<br>ACTGTGAGGAGAGAAAGTACTTTGAGGATGCGCAGTGA
CarB	ATGTCCAAGAAACACATTGTCATTATCGGTGCTGGCGTGGGTGGCAC<br>GGCTACAGCTGCTCGTTTGGCCCGCGAAGGCTTCAAGGTCACTGTGG<br>TGGAGAAAAACGACTTTGGTGGCGGCCGATGCTCCTTGATCCATCAC<br>CAGGGCCATCGCTTTGATCAGGGCCCGTCGCTCTACCTGATGCCCAA<br>GTACTTTGAGGACGCCTTTGCCGATCTGGACGAGCGCATTCAAGACC<br>ACCTGGAGCTGCTGCGATGCGACAACAACTACAAGGTGCACTTTGAC<br>GACGGTGAGTCGATCCAGCTGTCGTCTGACTTGACACGCATGAAGGC

	TGAATTGGACCGCGTGGAGGGCCCCCTTGGTTTTGGCCGATTCCTGG<br>ATTTCATGAAAGAGACACACATCCACTACGAAAGCGGCACCCTGATT<br>GCGCTCAAGAAGAATTTCGAATCCATCTGGGACCTGATTCGCATCAA<br>GTACGCTCCAGAGATCTTTCGCTTGCACCTGTTTGGCAAGATCTACGA<br>CCGCGCTTCCAAGTACTTCAAGACCAAGAAGATGCGCATGGCATTCA<br>CGTTTCAGACCATGTATATGGGCATGTCGCCCTACGATGCGCCTGCTG<br>TCTACAGCCTGTTGCAGTACACCGAGTTCGCTGAAGGCATCTGGTATC<br>CCCGTGGCGGCTTCAACATGGTGGTTCAGAAGCTAGAGGCGATTGCA<br>AAGCAAAAGTACGATGCCGAGTTTATCTACAATGCGCCTGTTGCCAA<br>GATTAACACCGATGATGCCACCAAACAAGTGACAGGTGTAACCTTGG<br>AAAATGGCCACATCATCGATGCCGATGCGGTTGTGTGTAACGCAGATC<br>TGGTCTATGCTTATCACAATCTGTTGCCTCCCTGCCGATGGACGCAAA<br>ACACACTGGCTTCCAAGAAATTGACGTCTTCTTCCATTTCCTTCTACT<br>GGTCCATGTCCACCAAGGTGCCTCAATTGGACGTGCACAACATCTTTT<br>TGGCCGAGGCTTATCAGGAGAGCTTTGACGAAATCTTCAAGGACTTT<br>GGCCTGCCTTCTGAAGCCTCCTTCTACGTCAATGTGCCCTCTCGCATC<br>GATCCTTCTGCTGCTCCCGACGGCAAGGACTCTGTCATTGTCTTGGTG<br>CCTATTGGTCATATGAAGAGCAAGACGGGCGATGCTTCCACCGAGAA<br>CTACCCGGCCATGGTGGACAAGGCACGCAAGATGGTGCTGGCTGTGA<br>TTGAGCGTCGTCTGGGCATGTCGAATTTCGCCGACTTGATTGAGCATG<br>AGCAAGTCAATGATCCCGCTGTATGGCAGAGCAAGTTCAATCTGTGG<br>AGAGGCTCAATTCTGGGTTTGTCTCATGATGTGCTTCAGGTGCTGTGG<br>TTCCGTCCCAGCACAAAGGATTCTACCGGTCGTTATGATAACCTATTC<br>TTTGTGGGTGCAAGCACGCATCCCGGAACTGGTGTTCCCATTGTCCTT<br>GCAGGAAGCAAGCTCACCTCTGACCAAGTTGTCAAGAGCTTTGGAA<br>AGACGCCCAAGCCAAGAAAGATCGAGATGGAGAACACGCAAGCACC<br>TTTGGAGGAGCCTGATGCTGAATCGACATTCCCTGTGTGGTTCTGGTT<br>GCGCGCTGCCTTTTGGGTCATGTTTATGTTCTTTTACTTCTTCCCTCAA<br>TCCAATGGCCAAACGCCCGCATCTTTTATCAATAATTTGTTACCTGAA<br>GTATTCCGCGTTCATAACTCTAATGTCATTTAA
CarRP	ATGCTGCTCACCTACATGGAAGTCCACCTCTACTACACGCTGCCTGTG<br>CTGGGCGTCCTGTCCTGGCTGTCGCGGCCGTACTACACAGCCACCGA<br>TGCGCTCAAATTCAAATTTCTGACACTGGTTGCCTTCACGACCGCCTC<br>CGCCTGGGACAACTACATTGTCTACCACAAGGCGTGGTCCTACTGCC<br>CCACCTGCGTCACCGCTGTCATTGGCTACGTGCCCTTGGAGGAGTAC<br>ATGTTCTTCATCATCATGACTCTGTTGACCGTGGCATTCACCAATCTGG<br>TGATGCGCTGGCACCTGCACAGCTTCTTTATCAGGCCTGAAACGCCC<br>GTCATGCAGTCCGTCCTGGTCCGTCTTGTCCCCATAACAGCCTTATTA<br>ATCACTGCATACAAGGCTTGGCATTTGGCGGTCCCTGGAAAGCCACT<br>GTTCTACGGATCATGCATTTTGTGGTACGCCTGTCCGGTTTTGGCCTTA<br>TTGTGGTTTGGTGCTGGCGAGTACATGATGCGTCGTCCGCTGGCGGT<br>GCTCGTGTCCATTGCGCTGCCCACGCTGTTTCTCTGCTGGGTCGATGT<br>CGTCGCTATTGGCGCCGGCACATGGGACATTTCGCTGGCCACAAGCA<br>CCGGCAAGTTCGTCGTGCCCCACCTGCCCGTGGAGGAATTCATGTTC<br>TTTGCGCTAATTAATACCGTTTTGGTATTTGGTACGTGTGCGATCGATC<br>GCACGATGGCGATCCTCCACCTGTTCAAAAACAAGAGTCCTTATCAG<br>CGCCCATACCAGCACAGCAAGTCGTTCCTCCACCAGATCCTCGAGAT<br>GACCTGGGCCTTCTGTTTACCCGACCAAGTGCTGCATTCAGACACATT<br>CCACGACCTGTCCGTCAGCTGGGACATCCTGCGCAAGGCCTCCAAGT<br>CCTTTTACACGGCCTCTGCTGTCTTTCCCGGCGACGTGCGCCAAGAG

	CTCGGTGTGCTATACGCCTTTTGCAGAGCCACGGACGATCTCTGCGAC<br>AACGAGCAGGTCCCTGTGCAGACGCGAAAGGAGCAGCTGATACTGA<br>CACATCAGTTCGTCAGCGATCTGTTTGGCCAAAAGACAAGCGCGCCG<br>ACTGCCATTGACTGGGACTTTTACAACGACCAACTGCCTGCCTCGTG<br>CATCTCTGCCTTCAAGTCGTTCACCCGTTTGCGCCATGTGCTGGAAGC<br>TGGAGCCATCAAGGAACTGCTCGACGGGTACAAGTGGGATTTGGAGC<br>GTCGCTCCATCAGGGATCAGGAGGATCTCAGATATTACTCAGCTTGTG<br>TCGCCAGCAGTGTTGGTGAAATGTGCACTCGCATCATACTGGCCCAC<br>GCCGACAAGCCCGCCTCCCGCCAGCAAACACAGTGGATCATTCAGCG<br>TGCGCGTGAAATGGGTCTGGTACTCCAATATACAAACATTGCAAGAG<br>ACATTGTCACCGACAGCGAGGAACTGGGCAGATGCTACCTGCCTCAG<br>GATTGGCTTACCGAGAAGGAGGTGGCGCTGATTCAAGGCGGCCTTGC<br>CCGAGAAATTGGCGAGGAGCGATTGCTCTCACTGTCGCATCGCCTCA<br>TCTACCAGGCAGACGAGCTCATGGTGGTTGCCAACAAGGGCATCGAC<br>AAGCTGCCCAGCCATTGTCAAGGCGGCGTGCGTGCGGCCTGCAACGT<br>CTATGCTTCCATTGGCACCAAGCTCAAGTCTTACAAGCACCACTATCC<br>CAGCAGAGCACATGTCGGCAATTCGAAACGAGTGGAAATTGCTCTTC<br>TTAGCGTATACAACCTTTACACCGCGCCAATTGCGACTAGTAGTACCA<br>CACATTGCAGACAGGGAAAAATGAGAAATCTAAATACCATTTAA

Supplementary Data Table 3: Plasmids used in this study

**Plasmid**	**Description**	**Source**
pJMP62-Nat-FDHbs-Tef	JMP-series *Y. lipolytica* storage plasmid with Tef promoter and codon optimised FDHbs, NAT added	This study
pZNS1.2-SOD2-Tef	Yalicraft-series *Y. lipolytica* storage plasmid with Tef promoter and SOD2	This study
pZNS1.2-SOD3-Tef	Yalicraft-series *Y. lipolytica* storage plasmid with Tef promoter and SOD3	This study
pZNS1.2-GSR1-Tef	Yalicraft-series *Y. lipolytica* storage plasmid with Tef promoter and GSR1	This study
pCarT-HyG	JMP-series storage plasmid with Tef8UAS promoters and CarB / CarRP / GGS1	This study
pYatK001	Storage vector for genes	Yuzbashev et al. 2020
pTOPO-Tef	Storage vector for Tef promoter	This study
pTOPO-LipT1	Storage vector for Lip Terminator	This study
pJMP62	JMP-series *Y. lipolytica* storage plasmid with Tef promoter and codon optimised FDHbs	This study
pZNS-1.3	Expression vector for SOD2/SOD3/GSR1	This study

Supplementary Data Table 4: Primers used in this study

**Description**	**Sequence**

Tef promoter F screening primer	TCTCCTTGTCAACTCACACC
SOD2 assembly primer F	atgcGCTCTTCAATGCTCGCCCGAAACACTCTTC
SOD2 assembly primer R	cgatGCTCTTCTTTACTGGCCGTTGACAAACTTG
SOD3 assembly primer F	atgcGCTCTTCAATGCATGGTCTCAATCAGTCAG
SOD3 assembly primer R	cgatGCTCTTCTTTAGTACTTGGCCAGAGGTCG
GSR assembly primer F	atgcGCTCTTCAATGGCTTCTATCCCCCATTATGAC
GSR assembly primer R	cgatGCTCTTCTttaCTATCTCATGGTCACCAGCTC
FDHbs screening primer R	CATCCTTGTCAGAAGTCACAATG
SOD2 screening primer R	CACAGAGAGTGGTTGATGTGTC
SOD3 screening primer R	CCAGAAGATCAGCCTTGAG
GSR screening primer R	GTAGACCTTGACGTTCTCCTC
